# IL-10 Suppression of NK/DC Crosstalk Leads to Poor Priming of MCMV-Specific CD4 T Cells and Prolonged MCMV Persistence

**DOI:** 10.1371/journal.ppat.1002846

**Published:** 2012-08-02

**Authors:** Sanja Mandaric, Senta M. Walton, Thomas Rülicke, Kirsten Richter, Mathilde J. H. Girard-Madoux, Björn E. Clausen, Antonija Zurunic, Masahito Kamanaka, Richard A. Flavell, Stipan Jonjic, Annette Oxenius

**Affiliations:** 1 Institute of Microbiology, ETH Zurich, Zurich, Switzerland; 2 Institute of Laboratory Animal Science, University of Veterinary Medicine Vienna, Vienna, Austria; 3 Department of Immunology, Erasmus University Medical Center, Rotterdam, Netherlands; 4 Department of Histology and Embryology, Faculty of Medicine, University of Rijeka, Rijeka, Croatia; 5 Department of Immunobiology and Howard Hughes Medical Institute, Yale School of Medicine, New Haven, Connecticut, United States of America; Oregon Health Sciences University, United States of America

## Abstract

IL-10 is an anti-inflammatory cytokine that regulates the extent of host immunity to infection by exerting suppressive effects on different cell types. Herpes viruses induce IL-10 to modulate the virus-host balance towards their own benefit, resulting in prolonged virus persistence. To define the cellular and molecular players involved in IL-10 modulation of herpes virus-specific immunity, we studied mouse cytomegalovirus (MCMV) infection. Here we demonstrate that IL-10 specifically curtails the MCMV-specific CD4 T cell response by suppressing the bidirectional crosstalk between NK cells and myeloid dendritic cells (DCs). In absence of IL-10, NK cells licensed DCs to effectively prime MCMV-specific CD4 T cells and we defined the pro-inflammatory cytokines IL-12, IFN-γ and TNF-α as well as NK cell activating receptors NKG2D and NCR-1 to regulate this bidirectional NK/DC interplay. Consequently, markedly enhanced priming of MCMV-specific CD4 T cells in *Il10* ^−/−^ mice led to faster control of lytic viral replication, but this came at the expense of TNF-α mediated immunopathology. Taken together, our data show that early induction of IL-10 during MCMV infection critically regulates the strength of the innate-adaptive immune cell crosstalk, thereby impacting beneficially on the ensuing virus-host balance for both the virus and the host.

## Author Summary

Cytomegalovirus (CMV) infections are very widespread in mammalian hosts. Despite the fact that CMVs are usually well controlled by the immune system, they cause persistent life-long infection and have evolved a number of strategies to effectively modulate or hide from host immunity. Since the establishment of an immunosuppressive environment favors virus persistence, IL-10 is one of the host targets that CMVs actively use to tune the virus-host balance toward their own benefit, resulting in prolonged virus persistence and hence increased chance for horizontal transmission. Here, we delineate the mechanisms of how IL-10 exerts its powerful immune-suppressing function in the context of murine cytomegalovirus (MCMV) infection. We found that IL-10 specifically restrains the priming of MCMV-specific CD4 T cell responses by suppressing dendritic cell (DC) - natural killer cell (NK) crosstalk during acute MCMV infection. Target molecules mediating this bi-directional crosstalk between DCs and NK cells were the pro-inflammatory cytokines IL-12, IFN-γ and TNF-α as well as NK cell activating receptors NKG2D and NCR-1 and all of them were markedly suppressed by IL-10. A consequence resulting from this impeded DC-NK cross-talk by IL-10, leading to poor priming of MCMV-specific CD4 T cell responses was increased lytic CMV persistence and reduced development of host tissue damage. Our study indicates that early induction of IL-10 during MCMV infection critically regulates the strength of the innate-adaptive crosstalk, thereby imparting on the ensuing virus-host balance for the benefit of both the virus and the host.

## Introduction

Persistent viral infections are very widespread in mammalian hosts and are often associated with relevant clinical symptoms which can culminate in fatal disease [1]. The establishment of a persistent viral infection requires specific viral properties to achieve co-existence with potent antiviral defense mechanisms. Viruses have therefore evolved various strategies to effectively modulate or hide from host immunity. Herpes viruses, including cytomegalovirus (CMV), encode for immune evasion proteins, which either affect antigen presentation, innate immune signaling or modulate host cytokine responses [2]. IL-10 is an anti-inflammatory cytokine that plays an important role in the regulation of host immunity to infection [3]. It acts by multiple immunosuppressive modes, mainly affecting the expression of pro-inflammatory cytokines and chemokines, modulating the function of antigen-presenting cells and directly or indirectly suppressing effector T cell responses [4]. IL-10 is expressed during a number of persistent viral infections and might on the one hand favor viral replication and persistence by suppressing antiviral defense mechanisms, but on the other hand might also be beneficial for the host by limiting immunopathology in the setting of antigen persistence and active antiviral immunity. Herpes viruses such as CMV and EBV exploit the functions of cellular IL-10 by encoding for viral IL-10 homologues [5] and by interfering with the IL-10R signaling pathway. Consistent with an immunosuppressive role of the viral IL-10 homologue, a recent study in rhesus macaques infected with a CMV lacking the RhCMV homologue of IL-10 (rhcmv-IL-10) reported enhanced T and B cell immunity [6]. Mouse CMV does not encode for its own IL-10 homologue but instead uses cellular IL-10 to modulate host immunity. Thus, endogenous IL-10 promotes virus replication in the salivary gland, an important mucosal site of virus persistence, which is likely to facilitate horizontal transmission of the virus [7]. A role of IL-10 for promoting virus persistence has also been show in chronic LCMV Clone 13 infection, where the absence of IL-10 led to virus clearance and restored functionality of LCMV-specific CD8 T cells [8], [9]. Conversely with IL-10 being an important regulator of host immunity, it is implied to attenuate host tissue damage that could occur during unregulated antiviral immunity, in particular in the context of chronic infection. Indeed, MCMV-infected *Il10* ^−/−^ mice were reported to exhibit increased liver pathology and severity of MCMV disease, which was ameliorated by administration of recombinant IL-10 [10].

The impact of IL-10 during the early phase of persistent viral infections, in particular for the induction of adaptive immunity, has not yet been extensively evaluated. Here we investigate *in vivo* the role of cellular IL-10 during acute MCMV infection with specific emphasis on its regulation of innate-adaptive crosstalk. We show that IL-10 specifically limits MCMV-specific CD4 T cell responses, but not CD8 T cell responses, by suppressing myeloid CD8α^−^ DC and NK cell function. Via interfering with the NK/DC crosstalk, IL-10 suppressed the induction of CD4 T cell protective immunity, facilitated MCMV persistence, but prevented development of TNF-α mediated immunopathology. Taken together, our data establish that induction of IL-10 during acute CMV infection plays an important role in regulating the magnitude of innate-adaptive crosstalk, thus altering the balance between the virus and the host.

## Results

### *Il10* ^−/−^ mice show increased body weight loss and reduced viral loads during acute MCMV infection

Early control of MCMV replication in C57BL/6 mice is to a large extent mediated by NK cells, due to their robust activation via interaction of Ly49H activating receptor on NK cells and MCMV protein m157 expressed on the surface of infected cells [11]. However, it was shown that the majority of wild type (wt) strains of MCMV does not encode for the *m157* sequence that trigger NK cell responses through the engagement of the Ly49H receptor, indicating that the strong NK cell response mediated via Ly49H early upon MCMV infection is unlikely to be a very representative situation [12]. We therefore used an *m157* deletion mutant virus to avoid massive activation of NK cells via Ly49H. However, we corroborated our main findings with *m157*-sufficient wt MCMV (Fig. S8, S9).

**Figure 1:**
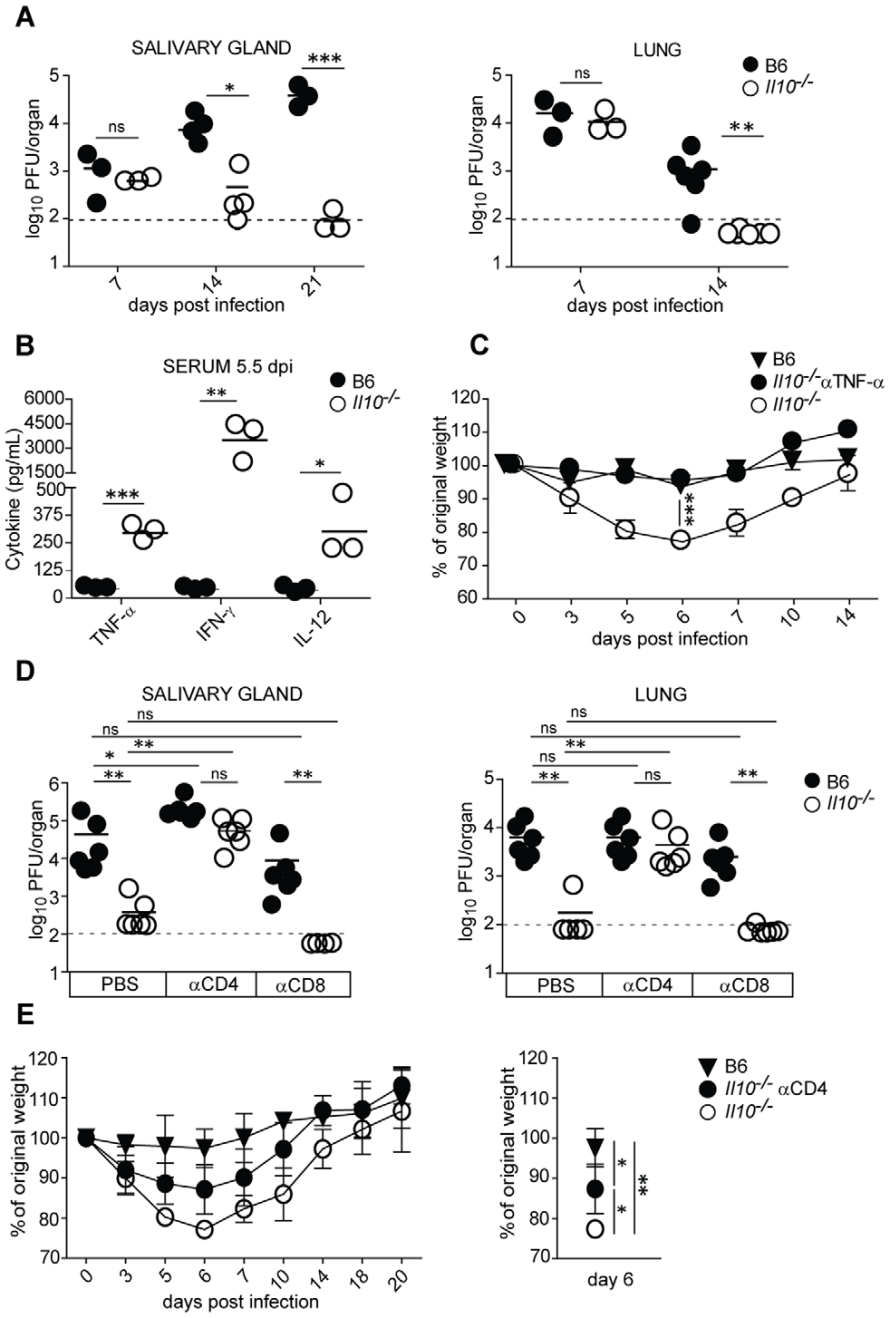
*Il10* ^−/−^ mice show reduced virus loads and increased body weight loss during acute MCMV infection. B6 and *Il10* ^−/−^ mice were infected i.v. with 5×10^6^ PFU *Δm157* MCMV. A) Virus titers on different days post infection were determined in lungs and salivary glands. Each symbol represents one individual mouse, horizontal line indicates the mean (n = 3–6, dashed line indicates detection limit). Data are representative of 3 independent experiments. B) IFN-γ, TNF-α and IL-12 protein concentrations were determined in the sera of B6 and *Il10* ^−/−^ mice at day 5.5 post infection by cytometric bead array (BD, Bioscience) and murine IL-12 ELISA Kit (Peprotech). Each symbol represents one individual mouse, horizontal line indicates the mean (n = 3). Data are representative of 2 independent experiments. C, E) Body weight change of B6 and *Il10* ^−/−^ mice was measured up to 14 or 20 days post infection. Change in percentage of body weight relative to day 0 is shown. One group of *Il10* ^−/−^ mice was treated with a neutralizing anti-TNF-α antibody (C) or depleted of CD4 T cells (E). Right panel in E indicates the same experiment showing significances for day 6 p.i. Data are representative of 3 independent experiments. Each symbol represents the mean of 3 mice per group, error bars indicate the standard deviation. D) Virus titers in lungs and salivary glands on day 14 post infection of B6 and *Il10* ^−/−^ mice with or without depletion of CD4 or CD8 T cells. Each symbol represents one individual mouse, horizontal line indicates the mean (n = 5–7, dashed line indicates detection limit). Pooled data from 2 independent experiments are shown. Statistical analysis was performed by 2-tailed unpaired student's t-test (* p<0.05, ** p<0.01, *** p<0.001).

Based on recently published data that induction of IL-10 during MCMV infection promotes virus persistence in the salivary glands and decreases latent viral loads [7], [13], we were interested to define the time point when IL-10 starts to influence the MCMV control in various organs. We found that MCMV-infected *Il10* ^−/−^ mice exhibited reduced virus loads already during acute infection (Figure 1A). While virus titers in the salivary gland and lungs were comparable with B6 mice at day 7 post infection (p.i.), they were considerably diminished at day 14 and 21 p.i. in *Il10* ^−/−^ mice. Elevated levels of pro-inflammatory cytokines IFN-γ and TNF-α were found at day 5.5 of infection in *Il10* ^−/−^ mice (Figure 1B). Furthermore, *Il10* ^−/−^ mice developed enhanced body weight loss early upon infection (Figure 1C), reaching the nadir between day 5 and 6 p.i., but recovered thereafter to B6 weight levels by day 14 p.i. To determine whether the increased body weight loss in *Il10* ^−/−^ mice was a consequence of excessive cytokine production, we neutralized TNF-α (Figure 1C), since neutralization of IFN-γ was reported to have no effect on the development of pathology in *Il10* ^−/−^ mice [14]. Notably, neutralization of TNF-α abolished body weight loss in *Il10* ^−/−^ mice, indicating that unregulated secretion of TNF-α mediated the development of pathology in *Il10* ^−/−^ mice (Figure 1C).

To address whether the enhanced control of viral replication in *Il10* ^−/−^ mice was caused by T cells, we used αCD4 and αCD8 depleting antibodies to eliminate the respective cell types (Figure 1D). We found that *Il10* ^−/−^ mice depleted of CD4 T cells exhibited significantly increased virus titers compared to PBS treated *Il10* ^−/−^ mice, indicating a pivotal role of CD4 T cells in the enhanced MCMV control in *Il10* ^−/−^ mice. In contrast, CD8 T cells were dispensable for increased anti-MCMV immunity in *Il10* ^−/−^ mice, since their depletion did not affect virus control. Moreover, CD4 T cell depleted *Il10* ^−/−^ mice showed reduced body weight loss compared to untreated *Il10* ^−/−^ mice (Figure 1E). Thus, we conclude that CD4 T cells are responsible for the enhanced MCMV control and contribute to body weight loss observed in *Il10* ^−/−^ mice. However, other cell types likely contribute to increased body weight loss in *Il10* ^−/−^ mice as well, since CD4 T cell depleted *Il10* ^−/−^ mice still lost more body weight than untreated B6 mice (Figure 1E).

Taken together, these data demonstrate that *Il10* ^−/−^ mice harbor reduced viral loads at the expense of increased body weight loss during the acute phase of MCMV infection due to the action of CD4 but not CD8 T cells. Hence, IL-10 has a dual role during the course of early MCMV infection; it inhibits the development of pathology, which is beneficial for the host, but at the same time it promotes lytic replication of the virus for prolonged time periods, which is beneficial for transmission of the virus.

### IL-10 differentially affects MCMV-specific CD4 *vs* CD8 T cell responses during acute infection

**Figure 2:**
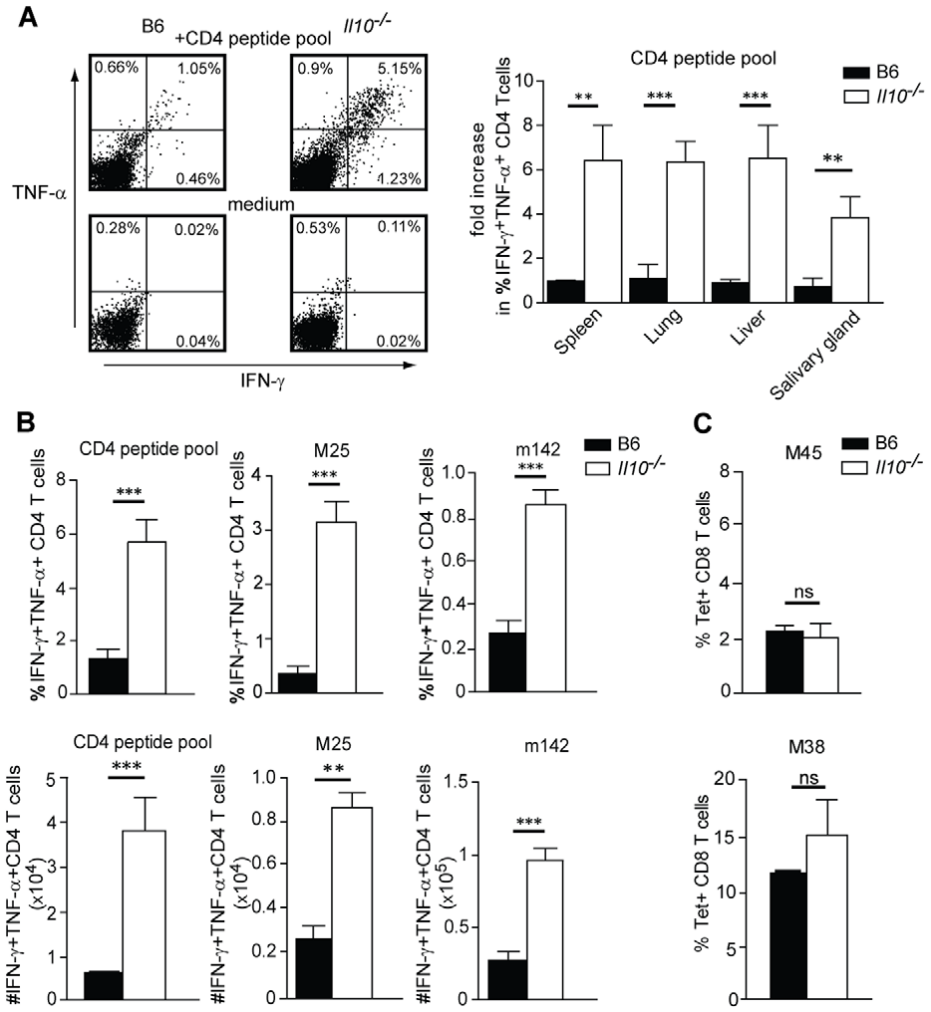
IL-10 differentially affects MCMV-specific CD4 vs CD8 T cell responses. B6 or *Il10* ^−/−^ mice were infected i.v. with 5×10^6^ PFU *Δm157* MCMV. Lymphocytes from spleen, lungs, liver and salivary gland (A) or lung (B) were isolated at day 14 post infection and *ex vivo* restimulated with appropriate peptides. A, B) CD4 T cells were restimulated with a pool of M14, m18, M25, M112, m139 and m142 peptides (CD4 peptide pool, A, B), or with M25 and m142 peptide alone (B). Fold increase in percentage of IFN-γ^+^ TNF-α^+^ peptide-specific CD4 T cells between *Il10* ^−/−^ and B6 mice (A, right panel, (n = 3), data are representative for at least 3 experiments, error bars indicate the standard deviation). (B) Total numbers (lower row) and percentages (upper row) of IFN-γ ^+^ TNF-α^+^ peptide-specific CD4 T cells from B6 and *Il10* ^−/−^ mice. C) Lung lymphocytes were isolated from infected mice and M45- or M38-specific CD8 T cells were quantified by tetramer staining (n = 3, data are representative from at least 3 experiments, error bars indicate the standard deviation). Statistical analysis was performed by 2-tailed unpaired student's t-test (* p<0.05, ** p<0.01, *** p<0.001).

To investigate in more detail the CD4 dependent regulation of virus control in *Il10* ^−/−^ mice, we analyzed the pool size and functionality of virus-specific CD4 and CD8 T cells upon MCMV infection. T cells were isolated from different organs and *ex vivo* restimulated with MCMV-derived CD4 and CD8 T cell peptide epitopes (Figure 2). We found that *Il10* ^−/−^ mice showed markedly increased frequencies and total numbers of MCMV-specific CD4 T cells producing IFN-γ and TNF-α at day 14 p.i. This massive increase of MCMV-specific CD4 T cells was apparent in all organs tested (Figure 2A, B).

In contrast, MCMV-specific CD8 T cell responses were not significantly affected in *Il10* ^−/−^ mice during acute infection. MCMV infection shapes the CD8 T cell response in a particular way, concomitantly driving the accumulation of CD8 T cells specific for certain viral peptides (inflationary responses) and inducing classical expansion/contraction kinetics of others (conventional responses). We analyzed the size of one representative CD8 T cell response of the conventional and inflationary type of MCMV-specific CD8 T cells [15]. We found that conventional (specific for the M45 epitope) as well as inflationary (specific for the M38 epitope) CD8 T cell responses were comparable in MCMV-infected *Il10* ^−/−^ and B6 mice (Figure 2C, Figure S1). Taken together, these data establish that IL-10 differentially affects T cell populations during the acute phase of infection; while it profoundly inhibits virus-specific CD4 T cell response it has only a minor impact on MCMV-specific CD8 T cell responses.

### Early induction of IL-10 suppresses CD8α^−^CD11b^+^ DC maturation and leads to poor CD4 T cell priming during MCMV infection

Since we observed a major impact of IL-10 on the virus-specific CD4 T cell response, we were interested to identify whether IL-10 is acting directly on CD4 T cells or indirectly through the modulation of the quality and phenotype of APCs. We addressed the first possibility by generating mixed bone marrow (BM) chimeras in which only CD4 T cells were either deficient or sufficient for the IL-10Rβ or in which all cells lacked IL-10Rβ expression. While the latter group exhibited massively increased numbers MCMV-specific CD4 T cells upon infection and showed increased control of viral replication, CD4 T cell responses in the presence or absence of IL-10Rβ selectively on CD4 T cells resulted in similarly low frequencies of MCMV-specific CD4 T cells as in presence of IL-10Rβ on CD4 T cells, indicating that direct IL-10 signaling on CD4 T cells does not constrain the size of the MCMV-specific CD4 T cell pool (Figure S2 and Text S1). However, viral titers in the salivary gland were to some extent reduced by selective absence of IL-10Rβ on CD4 T cells, suggesting a role for IL-10R signaling in CD4 T cells in this particular tissue (Figure S2).

**Figure 3:**
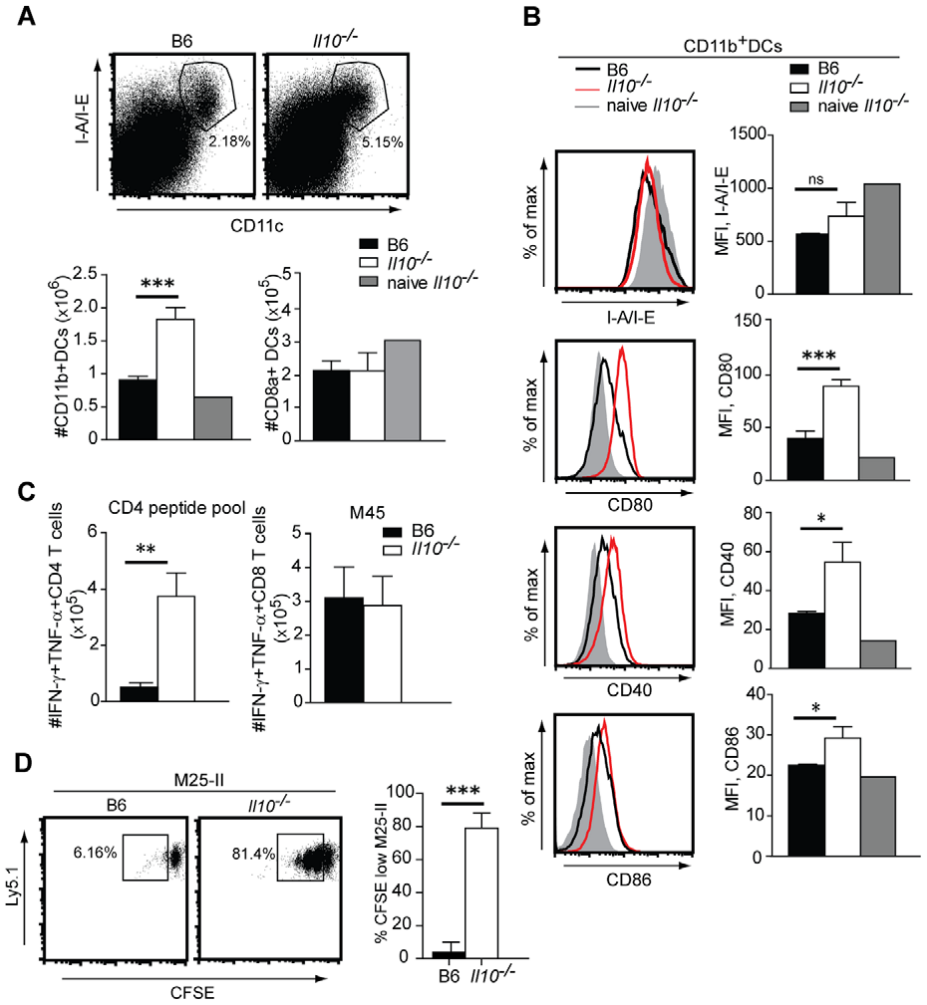
IL-10 alters the phenotype and function of DCs. B6 and *Il10* ^−/−^ mice were infected with 5×10^6^ PFU *Δm157* MCMV. A) Splenocytes from infected mice were isolated at day 5.5 p.i. Representative FACS plots showing CD11c and I-A/I-E stainings of total spleen leukocytes (upper row). Total numbers of CD11c^+^CD8α^−^CD11b^+^MHCII^+^B220^−^ DCs and CD11c^+^CD8α^+^MHCII^+^B220^−^ DCs from infected B6 and *Il10* ^−/−^ mice and naive *Il10* ^−/−^ mice are shown (lower row, n = 3 except for naive *Il10* ^−/−^ mice n = 1; data are representative for at least 3 experiments, error bars indicate the standard deviation). B) Expression levels of I-A/I-E and costimulatory molecules CD80, CD86 and CD40. Plots are gated on CD11c^+^CD8α^−^MHCII^+^B220^−^DC population at day 5.5 p.i. are shown (n = 3, error bars indicates standard deviation, data are representative from at least 3 experiments). Representative FACS pots (left column) and summary of MFI data (right column). C) Lung lymphocytes were isolated at day 5.5. p.i. and CD4 T cells were restimulated with a pool of M14, m18, M25, M112, m139 and m142 peptides (CD4 peptide pool) and CD8 T cells were restimulated with M45 peptide. Total numbers of IFN-γ^+^ TNF-α^+^ peptide specific CD4 and CD8 T cells are shown (n = 3, error bars indicate standard deviation, data are representative from at least 3 experiments). D) Splenic CD11c^+^ cells isolated at day 3.5 p.i. from B6 and *Il10* ^−/−^ mice were enriched by MACS, loaded with M25 peptide and incubated with the naive MACS purified CFSE labeled TCR transgenic CD4 T cells specific for the M25 protein (M25-II cells) for 3 days. The frequencies of CFSE^low^ M25 II cells are indicated in the representative FACS plot. (n = 3 triplicates of respective cell cultures, data are representative of 3 independent experiments). Statistical analysis was performed by 2-tailed unpaired student's t-test (* p<0.05, ** p<0.01, *** p<0.001).

Next, we sought to investigate whether the absence of IL-10 modulates the phenotype and function of APCs and thereby enhances the priming of virus-specific CD4 T cells. The absence of IL-10 led to an increase of the CD11b^+^CD11c^+^MHCII^+^B220^−^ myeloid DC population, which was most pronounced at day 5.5 p.i. (Figure 3A). In contrast, the lack of IL-10 had no effect on the CD8α^+^CD11c^+^MHCII^+^B220^−^ DC compartment, which was reduced in numbers compared to naive control mice, but to the same extent in B6 and *Il10* ^−/−^ mice. Consistent with this early expansion of myeloid DCs, we observed a greatly increased virus-specific CD4 T cell response already at day 5.5 p.i. (Figure 3C). In contrast, corroborating our results from day 14 p.i. (Figure 2C), virus-specific CD8 T cell responses were not affected by the lack of IL-10 at day 5.5 p.i. (Figure 3C). Since the CD8α^+^ DC subset is instrumental for priming of MCMV-specific CD8 T cells [16], the fact that IL-10 does not influence this DC population could explain why absence of IL-10 does not impact on MCMV-specific CD8 T cell responses (Figure 3A).

Next, we analyzed the expression levels of MHCII and costimulatory molecules on the surface of myeloid DCs (Figure 3B). We observed a downregulation of MHCII expression on CD8α^−^CD11b^+^CD11c^+^ DCs in infected mice compared to naive controls, which was the similar for B6 and *Il10* ^−/−^ mice. In contrast, expression levels of CD80, CD86 and CD40 costimulatory molecules on CD8α^−^CD11b^+^CD11c^+^ DCs were consistently higher in *Il10* ^−/−^ mice compared to their B6 counterparts. These effects were most pronounced at day 5.5 p.i. but some of the differences were already apparent at day 3.5 p.i. (data not shown). Of note, expression levels of costimulatory molecules on CD8α^+^CD11c^+^ DCs were comparable in B6 and *Il10* ^−/−^ mice, further supporting our observation that CD8 T cell responses are not affected by the presence or absence of IL-10 (Figure S3). Finally, to assess the functional consequences of these phenotypic differences in myeloid DCs of *Il10* ^−/−^ mice, we directly analyzed the capacity of DCs isolated from MCMV-infected B6 or *Il10* ^−/−^ mice to prime MCMV-specific CD4 T cells *in vitro* (Figure 3D and Text S1). To this aim, we used a monoclonal population of naive CD4 T cells specific for an immunodominant CD4 T cell epitope of the MCMV M25 protein [17] isolated from the spleen of a novel TCR transgenic mouse line named M25-II (Figure S4 and Text S1). DCs were isolated from B6 and *Il10* ^−/−^ mice at day 3.5 p.i., loaded with limiting amounts of M25 peptide and incubated with CFSE-labeled M25-II CD4 T cells. Proliferation was assessed by measuring CFSE dilution. Our results revealed that DCs isolated from MCMV-infected *Il10* ^−/−^ mice supported considerably enhanced priming capacity of TCR transgenic M25-II cells compared to DCs from MCMV-infected B6 mice. This was also the case for direct *ex vivo* antigen presenting DCs in absence of additional peptide loading *in vitro*, albeit the level of proliferation was smaller without additional peptide loading (data not shown).

**Figure 4:**
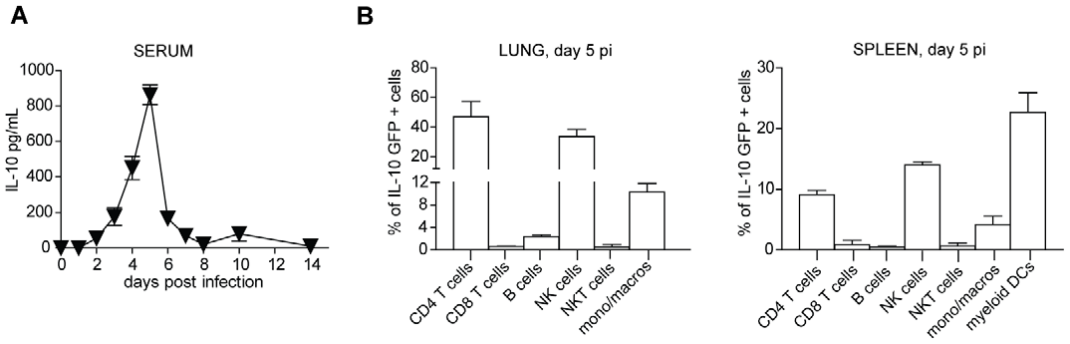
IL-10 is produced early upon MCMV infection. B6 or *Il10* GFP reporter (Tiger) mice were infected with 5×10^6^ PFU *Δm157* MCMV. A) IL-10 protein concentration was determined in the sera of infected B6 mice by IL-10 ELISA Set (BD, Biosciences) at indicated time points (n = 3, data are representative of 2 independent experiments, error bars indicate the standard deviation). B) Lung and spleen lymphocytes were isolated from infected *Il10* GFP reporter (Tiger) mice and control littermates at day 5.5 p.i. Percentages of GFP^+^ cells (after substraction of background fluorescence from littermate controls) are shown for the indicated cell populations: CD4^+^ cells, CD8^+^ cells, B220^+^ cells (B cells), NK1.1^+^CD3ε^−^ cells (NK cells), NK1.1^+^CD3ε^+^ cells (NK T cells), CD11b^+^CD11c^−^LyG/C^−^ cells (monocytes/macrophages) and splenic CD11c^+^CD11b^+^MHCII^+^B220^−^ cells (myeloid DCs). (n = 3, data are representative of 2 independent experiments, error bars indicate the standard deviation).

Our observation that IL-10 shapes the potency of DCs to prime virus-specific CD4 T cells already by 3.5 days p.i. suggested that MCMV would induce early secretion of IL-10 in order to dampen innate immune responses and to attenuate DC function, eventually leading to impaired CD4 T cell priming. Indeed, IL-10 levels in the serum of infected mice were detectable early upon infection, reaching a peak at day 5 p.i. (Figure 4A). Using *Il10*-GFP reporter mice, we demonstrated that MCMV induces expression of IL-10 by various cell types, with CD4 T cells, NK cells, DCs and macrophages being the most prominent IL-10 producers (Figure 4B). In order to dissect the *in vivo* relevance of IL-10 secretion by particular cell types, we took advantage of mixed BM chimeras in which specific cell types were unable to produce IL-10 (Figure S5 and Text S1). Intriguingly, we identified CD11c^+^ cells and to a lesser extent macrophages/neutrophils, but not CD4 T cells, as the prominent *in vivo* source of IL-10 during early MCMV infection, leading to reduced MCMV-specific CD4 T cell responses and elevated virus load during acute MCMV infection (Figure S5).

**Figure 5:**
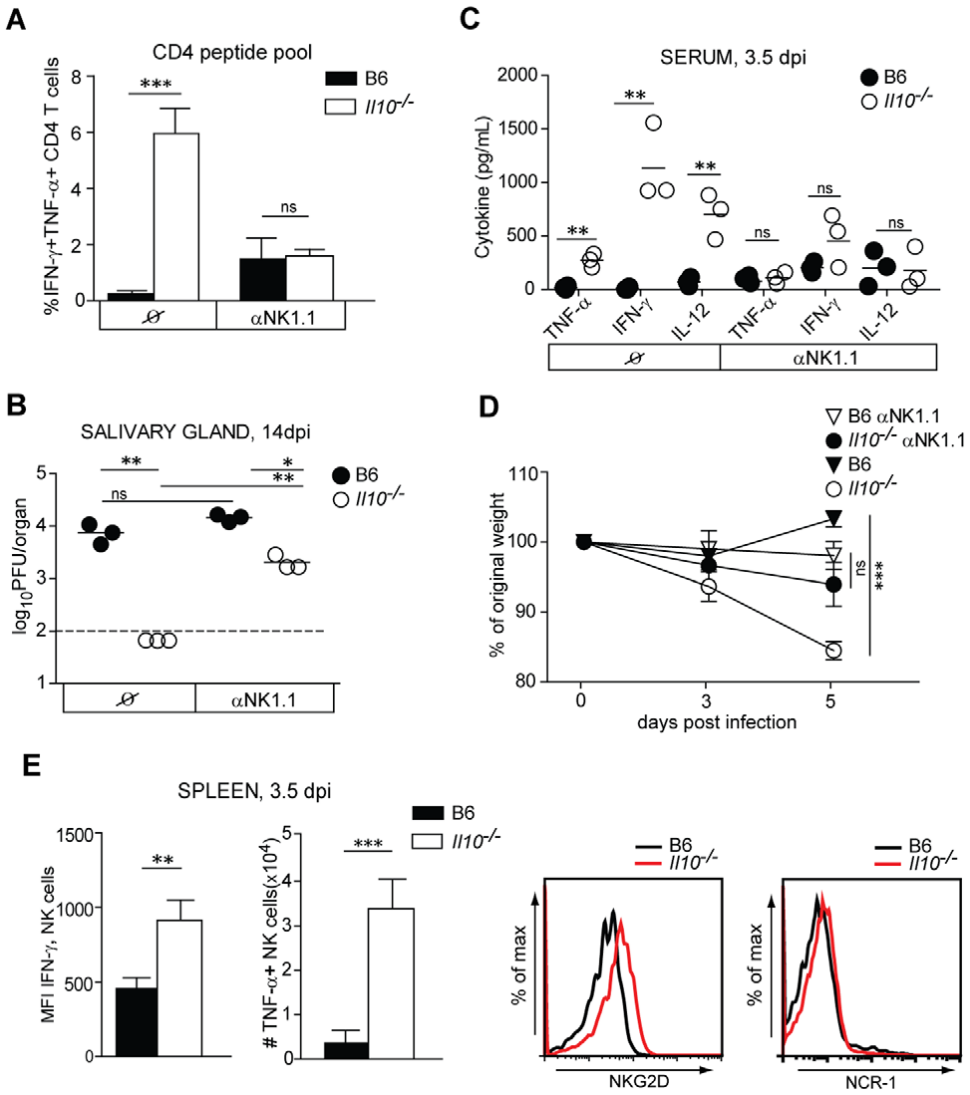
NK-like cells are responsible for enhanced MCMV-specific CD4 T cell response in *Il10* ^−/−^ mice. B6 and *Il10* ^−/−^ mice were infected with 5×10^6^ PFU *Δm157* MCMV. A–D) B6 and *Il10* ^−/−^ mice were either mock treated or depleted of NK-like cells using αNK1.1 (PK136) antibody. A) Lymphocytes from lungs were isolated at day 5.5. p.i. and *ex vivo* restimulated with a pool of M14, m18, M25, M112, m139 and m142 peptides (CD4 peptide pool). Percentages of IFN-γ^+^TNF-α^+^ peptide specific CD4 cells are shown (n = 3, error bars indicate standard deviation, data are representative from at least 3 experiments). B) Virus titers were determined in salivary glands at day 14 p.i. Each symbol represents one individual mouse, horizontal line indicates the mean, dashed line indicates detection limit (n = 3, data are representative of 2 independent experiments). C) IFN-γ, TNF-α and IL-12 protein concentrations were determined in the sera of B6 and *Il10* ^−/−^ mice at day 3.5 p.i. Each symbol represents one individual mouse, horizontal line indicates the mean (n = 3, data are representative of 2 independent experiments). D) Body weight change of B6 and *Il10* ^−/−^ mice was measured at the indicated time points. Changes in percentage of body weight relative to day 0 are shown. Each symbol represents the mean of 3 mice per group; vertical bars indicate the standard deviation. Data are representative of 3 independent experiments. E) Splenic NK1.1^+^CD3ε^−^ NK cells (NK) were isolated from B6 and *Il10* ^−/−^ mice at day 3.5 p.i. Total numbers of IFN-γ^+^ NK cells and MFI of IFN-γ^+^ in NK cells are shown (upper panel). Total numbers of TNF-α^+^ NK cells and expression levels of NKG2D and NCR-1 on NK cells are shown (lower panel). Error bars indicate standard deviation; n = 3, data are representative of 3 independent experiments. Statistical analysis was performed by 2-tailed unpaired student's t-test (* p<0.05, ** p<0.01, *** p<0.001).

Taken together, these data reveal that early induction of IL-10 during MCMV infection suppresses maturation of DCs, specifically targeting the myeloid CD8α^−^CD11b^+^CD11c^+^ subset, which results in poor CD4 T cell priming and prolonged lytic viral replication.

### NK-like cells are crucial enhancers of CD4 T cell priming during MCMV infection in *Il10* ^−/−^ mice

NK cells have recently been reported to influence the priming of MCMV-specific CD8 T cell responses by regulating the exposure of CD8 T cells to antigen-bearing DCs [18]. We were therefore interested to address the impact of NK cells on the priming of MCMV-specific CD4 T cell responses during MCMV infection in the absence or presence of IL-10.

To evaluate the impact of NK cells on MCMV-specific CD4 T cell priming in *Il10* ^−/−^ mice, we depleted NK-like cells using the αNK1.1 antibody. Strikingly, the frequencies of MCMV-specific CD4 T cells were massively reduced in NK-like cell depleted *Il10* ^−/−^ mice to levels, which were no longer different from NK-like cell depleted B6 mice (Figure 5A). Thus, the absence of NK-like cells completely abolished the massive increase of MCMV-specific CD4 T cell responses in *Il10* ^−/−^ as compared to B6 mice. Consistent with a critical contribution of NK-like cells for the priming of virus-specific CD4 T cell responses in *Il10* ^−/−^ mice, depletion of NK-like cells indeed partly abolished the protective effect of CD4 T cells observed in *Il10* ^−/−^ mice at day 14 post infection (Figure 5B), since αNK1.1 depleted *Il10* ^−/−^ mice showed a significantly increased viral burden compared to untreated *Il10* ^−/−^ mice (Figure S6A).

Furthermore, *Il10* ^−/−^ mice depleted of NK-like cells showed no significant increase in the levels of IFN-γ, TNF-α and IL-12 in the sera at day 3.5 p.i. compared to B6 controls (Figure 5C). To evaluate the impact of NK-like cells on the development of immunopathology in *Il10* ^−/−^ mice, we measured body weight loss in B6 and *Il10* ^−/−^ mice treated with αNK1.1 antibody and observed that *Il10* ^−/−^ mice depleted of NK-like cells were comparable to B6 controls (Figure 5D). These data indicate that NK-like cells are key contributors to the increased levels of pro-inflammatory cytokines as well as to the pathology observed in *Il10* ^−/−^mice.

Flow cytometric analysis of splenic NK cell populations revealed that *Il10* ^−/−^ mice exhibited decreased numbers of NK1.1^+^CD3^−^ cells upon MCMV infection (data not shown, [19]). However, although total numbers of IFN-γ^+^ NK1.1^+^CD3^−^ cells were unaltered in *Il10* ^−/−^ mice (not shown), NK cells from MCMV-infected *Il10* ^−/−^ mice showed increased per cell expression levels of IFN-γ already at day 3.5 p.i. compared to NK cells from MCMV-infected B6 mice (Figure 5E). Moreover, the numbers of TNF-α producing NK cells were massively increased in *Il10* ^−/−^ mice compared to B6 controls and NK cells from MCMV-infected *Il10* ^−/−^ mice displayed higher expression levels of the NKG2D and NCR-1 activating receptors compared to B6 controls (Figure 5E).

Taken together, these data demonstrate that in the absence of IL-10 NK-like cells provide critical help to virus-specific CD4 T cells, which results in better control of lytic virus replication in *Il10* ^−/−^ mice. Conversely, the hyper-activated phenotype of NK cells in *Il10* ^−/−^ mice results in early high levels of systemic pro-inflammatory cytokines, which either directly or in combination with MCMV-specific CD4 T cell-derived cytokines promote development of pathology.

### NK-like cells are the main contributors promoting maturation of DCs in *Il10* ^−/−^ mice during MCMV infection

**Figure 6:**
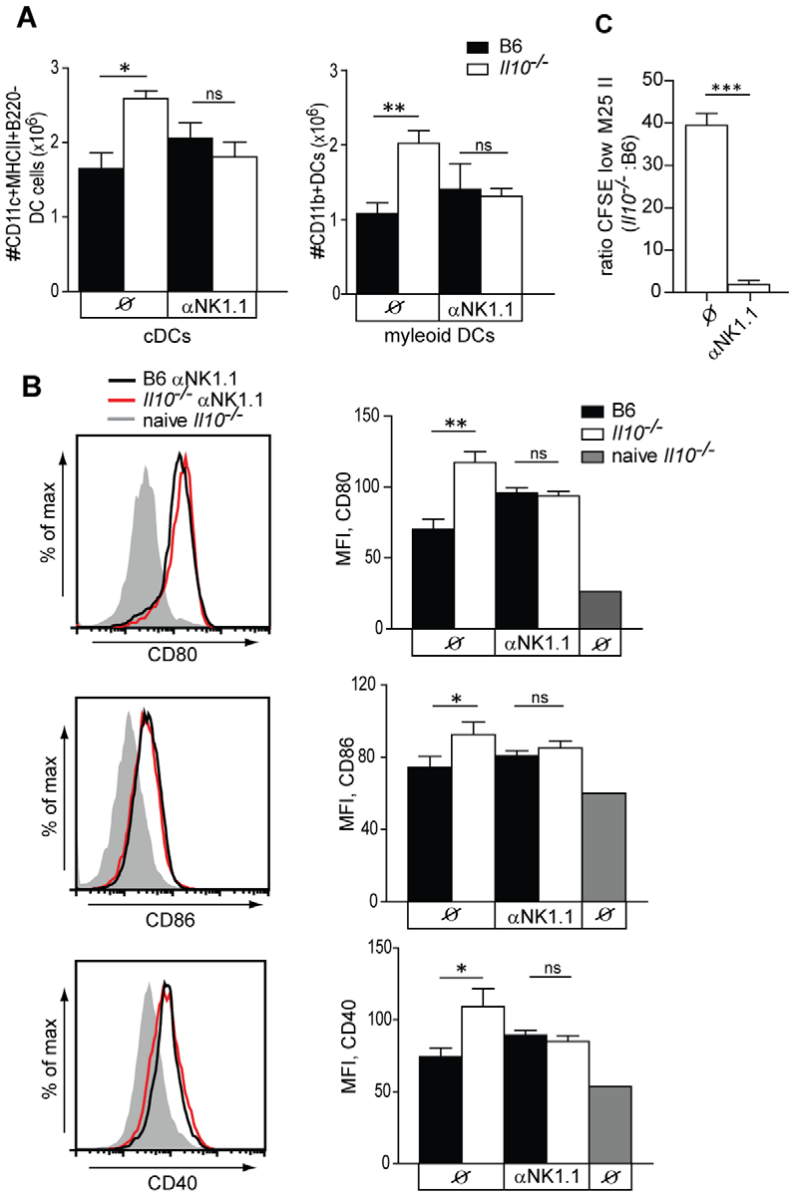
NK-like cells promote DC maturation in *Il10* ^−/−^ mice. B6 and *Il10* ^−/−^ mice were infected with 5×10^6^ PFU *Δm157* MCMV and were either mock treated or depleted of NK-like cells using αNK1.1 (PK136) antibody. A) Splenocytes from infected mice were isolated at day 5.5 post infection. Total numbers of CD11c^+^MHCII^+^B220^−^ DCs and CD11c^+^CD11b^+^MHCII^+^B220^−^ DCs from infected B6 and *Il10* ^−/−^ mice and naive *Il10* ^−/−^ mice are shown (n = 3, error bars indicates standard deviation, data are representative of 3 experiments). B) Expression levels of MHCII and costimulatory molecules CD80, CD86 and CD40 at day 5.5 p.i. Plots are gated on CD11c^+^CD11b^+^MHCII^+^B220^−^ DCs. Representative FACS pots (left column) and summary of MFI data (right column, n = 3, error bars indicates standard deviation, data are representative of 3 experiments). C) Splenic CD11c^+^ cells isolated at day 3.5 p.i. from NK-like depleted or undepleted B6 and *Il10* ^−/−^ mice were enriched by MACS, loaded with M25 peptide and incubated with MACS purified naive CFSE-labeled TCR transgenic M25-II CD4 T for 3 days. The ratio between CFSE^low^ M25-II cells in cultures with *Il10* ^−/−^ and B6 DCs is shown (n = 3 triplicates of respective cell cultures, data are representative of 2 independent experiments). Statistical analysis was performed by 2-tailed unpaired student's t-test (* p<0.05, ** p<0.01, *** p<0.001).

Next we analyzed the mechanism by which NK cells promote MCMV-specific CD4 T cell responses in the absence of IL-10. To this end, we first determined the DC phenotype and priming capacity in the absence of NK-like cells. *Il10* ^−/−^ and B6 mice were depleted of NK-like cells and splenic DCs were analyzed by flow cytometry at day 5.5 p.i. Increased numbers of splenic myeloid DCs observed in *Il10* ^−/−^ mice were not seen in the absence of NK-like cells (Figure 6A). Furthermore, expression levels of costimulatory molecules on the surface of DCs from αNK1.1 treated *Il10* ^−/−^ and B6 mice were determined by flow cytometry (Figure 6B). Interestingly, when NK-like cells were depleted from *Il10* ^−/−^ mice, no differences were observed any longer in the expression levels of costimulatory molecules compared to αNK1.1 treated B6 mice. Thus, in the absence of IL-10, NK-like cells are the main contributors promoting maturation of DCs.

Next, the CD4 T cell priming capacity of DCs isolated from MCMV-infected mice in the absence of NK-like cells was tested. DCs were isolated from MCMV-infected B6 and *Il10* ^−/−^ mice that had been treated or not with αNK1.1 antibody and incubated with naive CFSE labeled TCR transgenic M25-II CD4 T cells. The analysis of CFSE dilution revealed that the marked difference in the priming capacity of naive M25-II CD4 T cells between B6 and *Il10* ^−/−^ mice was no longer observed when DCs from αNK1.1 treated mice were used as APCs (Figure 6C and Text S1).

### Unleashed NK/DC crosstalk drives the efficiency of CD4 T cell priming in *Il10* ^−/−^ mice

**Figure 7:**
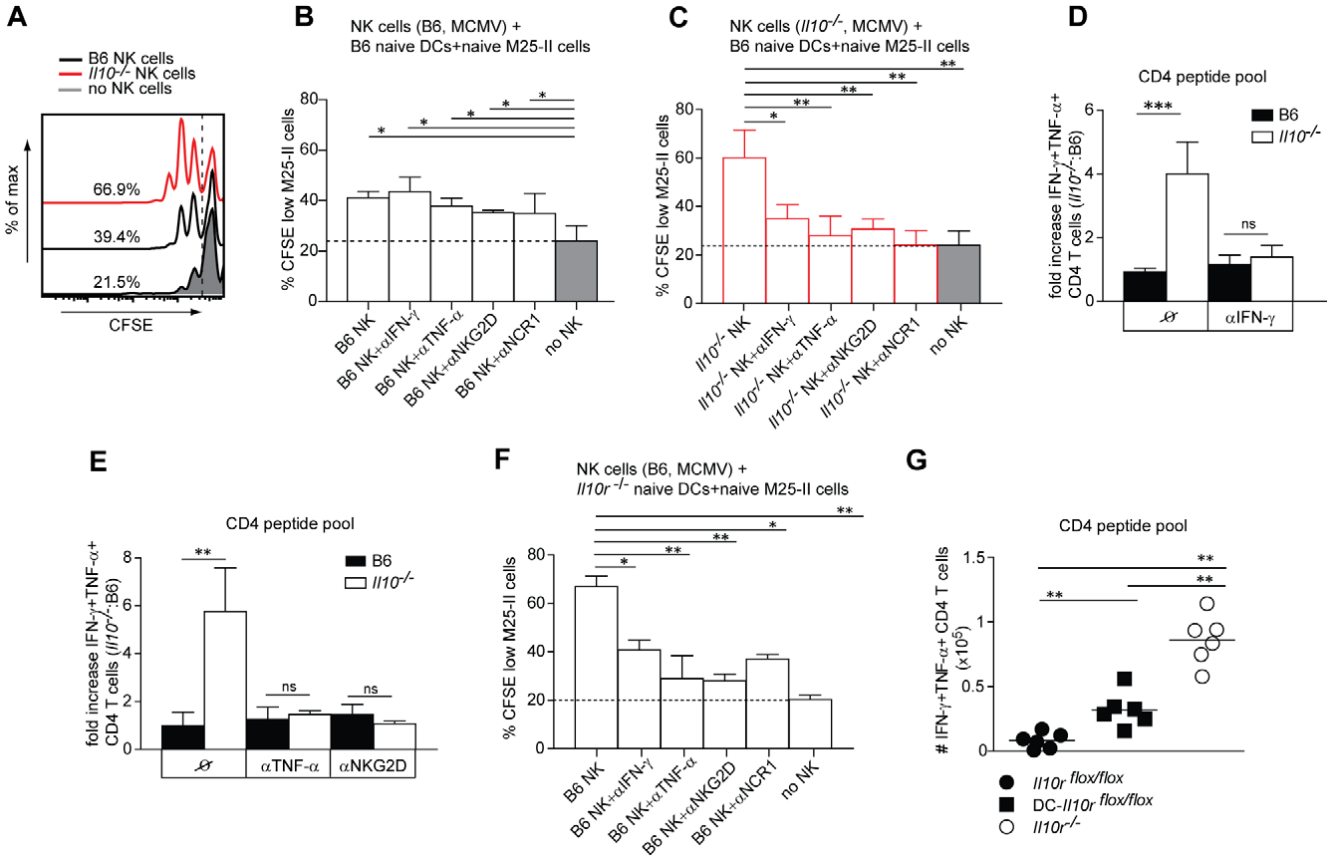
Unleashed NK/DC crosstalk promotes CD4 T cell priming in *Il10* ^−/−^ mice. B6 and *Il10* ^−/−^ mice were infected with 5×10^6^ PFU *Δm157* MCMV. A–C,F) DX5^+^CD3^−^ (NK) cells were isolated from B6 and *Il10* ^−/−^ mice by MACS at day 3.5 p.i. Splenic DCs were isolated from naive B6 (A–C) mice or naive *Il10r* ^−/−^ mice (F) by enrichment for CD11c^+^ cells. MCMV-specific CD4 T cells were isolated by MACS from spleens of naive M25-II mice and labeled with CFSE. CD11c^+^ cells were loaded with M25 peptide and co-cultured with CFSE-labeled M25-II cells without (no NK) or with addition of DX5^+^CD3^−^ (NK) cells isolated from B6 (A, B, F) or *Il10* ^−/−^ (A, C) mice. As indicated, blocking antibodies for IFN-γ, TNF-α, NKG2D and NCR-1 were added to cultures. The frequencies of CFSE^low^ M25 II cells are shown. (n = 3, data are representative of 3 independent experiments). Dotted lines indicate the mean level of M25-II CFSE dilution in cultures without NK cells (no NK). D, E) B6 and *Il10* ^−/−^ mice were treated *in vivo* with αIFN-γ antibodies at days 3 and 4 p.i. (D); with αTNF-α and αNKG2D antibodies at days 0, 3, 4 p.i. (E). Lymphocytes from lungs of infected mice were isolated at day 5.5 p.i. and *ex vivo* restimulated with a pool of M14, m18, M25, M112, m139 and m142 peptides (CD4 peptide pool). Fold increase (D, E) of IFN-γ^+^ TNF-α^+^ peptide specific CD4 cells between *Il10* ^−/−^ and B6 mice is shown (n = 3, error bars indicates standard deviation, data are representative for 3 experiments). G) DC- *Il10r* ^flox/flox^, *Il10r* ^flox/flox^ and *Il10r* ^−/−^ mice were infected with 5×10^6^ PFU *Δm157* MCMV. Lymphocytes from lungs were restimulated with the CD4 peptide pool. Total numbers of IFN-γ^+^ TNF-α^+^ peptide-specific CD4 cells are shown. (n = 3, data are pooled from 2 independent experiments). Statistical analysis was performed by 2-tailed unpaired student's t-test (* p<0.05, ** p<0.01, *** p<0.001).

Having shown that IL-10 suppresses the ability of NK-like cells to promote DC maturation and their CD4 T cell priming capacity, we aimed at identifying the underlying mechanism by which IL-10 dampens NK/DC crosstalk and priming of CMV-specific CD4 T cells. Previous reports have documented that NK cells can interact with DCs to shape the course of the innate as well as adaptive immune response [20]. This NK/DC crosstalk leads to reciprocal activation, which depends on cytokines and/or membrane receptor engagement. NK cell derived IFN-γ and TNF-α play important roles in promoting IL-12 production by DCs. Conversely, DC secretion of IL-12 and IL-18 enhances cytokine production of NK cells [21]. Moreover, NK/DC cell to cell contact has been implied to be important in the bidirectional cross-talk between these cell types. To identify the factors involved in NK/DC interactions that could promote CD4 T cell priming in the absence of IL-10, we established an *in vitro* co-culture system with purified populations of NK cells, DCs and virus-specific CD4 T cells. NK cells were purified from B6 and *Il10* ^−/−^ mice at day 3.5 p.i. and co-cultured with M25 peptide-loaded DCs isolated from naive mice together with naive CFSE-labeled TCR transgenic M25-II CD4 T cells. This experimental set-up, where only NK cells were isolated from MCMV infected mice, allowed us to directly dissect whether NK cells impact on the capacity of DCs to prime virus-specific CD4 T cells. Indeed, DCs co-cultured with NK cells from MCMV infected *Il10* ^−/−^ mice showed an increased priming capacity of naive virus-specific CD4 T cells compared to DCs co-cultured with the same number of NK cells isolated from MCMV infected B6 mice (Figure 7A). Comparably increased proliferation of MCMV-specific CD4 T cells was obtained when DCs were co-cultured with NK cells from MCMV infected B6 mice in presence of an IL-10R blocking antibody (data not shown). To identify the factors involved in these NK/DC interactions we preformed the same *in vitro* co-cultures as described above, including neutralization of IFN-γ, TNF-α and blocking of activating NK cell receptors NKG2D and NCR-1. Interestingly, neutralization or blocking of any of those factors did not have an influence on CD4 T cell priming when NK cells had been isolated from infected B6 mice (Figure 7B). In contrast, when NK cells had been isolated from *Il10* ^−/−^ mice, neutralization of IFN-γ and TNF-α as well as blocking of NKG2D and NCR-1 significantly decreased the priming of virus-specific CD4 T cells (Figure 7C). Moreover, based on our *in vitro* results, we tested whether some of the factors which modulated NK/DC crosstalk *in vitro* would also promote MCMV-specific CD4 T cell priming and expansion *in vivo*. We therefore administered neutralizing αIFN-γ, αTNF-α and blocking αNKG2D antibodies (Figure 7 D, E). These treatments increased the viral loads to the same extent in B6 and *Il10* ^−/−^ mice, compared to mock treated mice at early time points (Figure S6B). Strikingly, the fold increase in frequencies of MCMV-specific CD4 T cell responses was no longer different between B6 and *Il10* ^−/−^ mice when IFN-γ or TNF-α were neutralized or NKG2D was blocked *in vivo* (Figure 7D, E). Of note, *in vivo* neutralization of IFN-γ and TNF-α also abrogated the increased expression levels of NKG2D and NCR-1 on NK cells normally observed in *Il10* ^−/−^ mice to levels indistinguishable from NK cells in B6 mice (data not shown), suggesting that increased levels of the pro-inflammatory cytokines IFN-γ and TNF-α in MCMV infected *Il10^−/−^* mice presumably trigger NK cell activation more efficiently compared to B6 controls, resulting in the upregulation of NKG2D and NCR-1 receptors on the surface of NK cells.

Next, we hypothesized that IL-10 produced by NK cells (Figure 4B) could be directly sensed by DCs which would result in impaired NK/DC crosstalk and would hence account for the poor CD4 T cell priming observed in B6 mice. In support of this hypothesis, when DCs were isolated from naive *Il10r* ^−/−^ mice and co-cultivated with NK cells from infected B6 mice together with naive CFSE-labeled M25 II CD4 T cells (Figure 7F), the proliferation of M25 II cells was much more pronounced compared to the situation where DC were isolated from naive B6 mice (Figure 7F and B). Furthermore, also in this situation neutralization of IFN-γ and TNF-α as well as blocking of NKG2D and NCR-1 decreased the proliferation of M25 II cells (Figure 7F). Finally, as we had previously shown that DC-derived IL-10 suppresses MCMV-specific CD4 T cell responses *in vivo* (Figure S5), we also tested in a similar co-culture setup whether IL-10 production by DCs influenced their CD4 T cell priming capacity. To this end, NK cells from MCMV infected B6 mice were co-incubated with naive DCs from B6 or *Il10* ^−/−^ mice and proliferation of M25 II cells was assessed. Consistent with the *in vivo* role of DC-derived IL-10 production, M25 II proliferation was enhanced in co-cultures with *Il10* ^−/−^ DCs (Figure S7), suggesting that DCs can produce IL-10 in response to NK cell derived factors induced by MCMV infection, and that this autocrine suppressive effect, in addition to IL-10 production by NK cells, may also play an important role in balancing the outcome for the virus-specific CD4 T cell priming *in vivo*.

To test whether DCs have to sense IL-10 *in vivo* to suppress MCMV-specific CD4 T cell responses, we infected mice that specifically lack IL-10Rα on CD11c^+^ DCs (DC-*Il10r* ^−/−^, [22], Figure 7G). Indeed, MCMV-specific CD4 T cell responses were increased in these mice compared to controls (*Il10r* ^flox/flox^). However, CD4 T cell responses were still more pronounced in total than in DC-specific *Il10r* ^−/−^ mice. These data suggest that DCs are one, but not the only cell type that senses and is regulated by IL-10. It is likely that NK cells themselves are as well amongst the earliest targets of IL-10 upon MCMV infection to curtail their activation and cytokine production.

Taken together, these data corroborate the importance of NK cells as promoters of DC activation and maturation and subsequent CD4 T cell priming in MCMV infected *Il10* ^−/−^ mice, namely by providing soluble mediators such as IFN-γ and TNF-α, as well by cell to cell contact with involvement of the NKG2D and NCR-1 receptor. In contrast, the intensity of NK/DC synergy is markedly diminished in the presence of IL-10 leading to poor CD4 T cell priming with both DC and NK cells presumably being the early and important targets as well as sources of IL-10.

**Figure 8:**
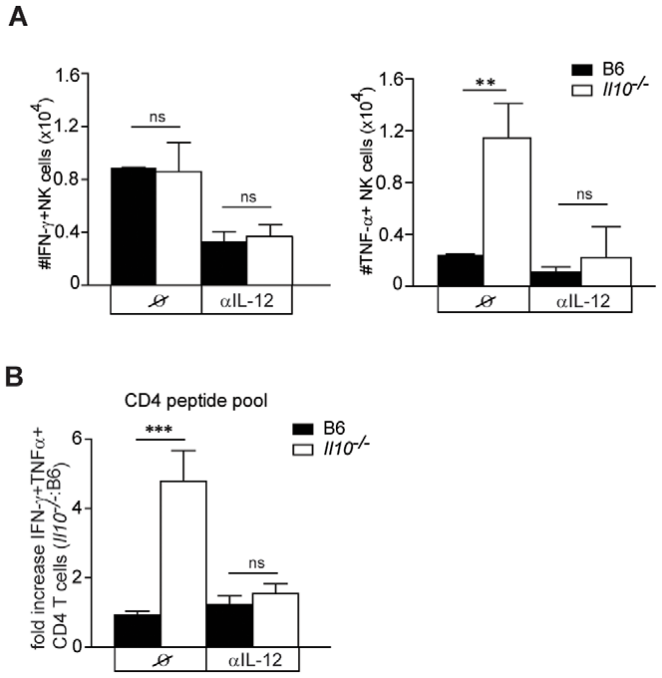
IL-12 contributes to enhanced CD4 T cell priming in *Il10* ^−/−^ mice. B6 and *Il10* ^−/−^ mice were infected with 5×10^6^ PFU *Δm157* MCMV and treated *in vivo* with αIL-12 antibody at days 0, 3, 4 p.i. A) Splenic NK1.1^+^CD3ε^−^ NK cells (NK) were isolated from B6 and *Il10* ^−/−^ mice at day 3.5 p.i. Total numbers of IFN-γ^+^ NK and TNF-α^+^ cells are shown (error bars indicate standard deviation; n = 3, data are representative of 2 independent experiments). B) Lymphocytes were isolated from lungs at day 5.5 p.i. and *ex vivo* restimulated with the CD4 peptide pool. Fold increase in IFN-γ^+^ TNF-α^+^c peptide specific CD4 T cells between *Il10* ^−/−^ and B6 is shown (n = 3, error bars indicates standard deviation, data are representative from 3 independent experiments). Statistical analysis was performed by 2-tailed unpaired student's t-test (** p<0.01, *** p<0.001, n.d. = not detected).

Conversely, it is known that DCs in turn can also promote activation of NK cells. IL-12 is one of the DC-derived cytokines that stimulates NK cell cytokine production. Moreover, IL-10 influences the IL-12/IFN-γ positive feedback loop during NK/DC crosstalk [23], but the consequences of these regulatory effects on the adaptive immune response during viral infections remain largely unknown. Since *Il10* ^−/−^ mice produced more IL-12 in response to MCMV and NK1.1. depleted *Il10* ^−/−^ mice showed decreased levels of IL-12 (Figure 5C), we sought to define the impact of IL-12 on the induction of NK cell and virus-specific CD4 T cell responses in the absence of IL-10 (Figure 8). αIL-12/p40 antibody-mediated neutralization of IL-12 reduced NK cell secretion of IFN-γ in B6 as well in *Il10* ^−/−^ mice, and secretion of NK cell derived TNF-α was markedly reduced in *Il10* ^−/−^ mice to the levels of B6 controls on day 5.5 p.i. (Figure 8A). Consistent with the role of NK cells in promoting MCMV-specific CD4 T cell priming in absence of IL-10, CD4 T cell responses were no longer increased in *Il10* ^−/−^ mice compared to B6 mice when IL-12 was neutralized *in vivo* (Figure 8B). Albeit neutralization of IL-12 led to increased viral loads at day 5.5 p.i., this increase in viral load was comparable in B6 and *Il10* ^−/−^ mice (Figure S6C). These data show that IL-12 plays a potent role in the activation of NK cells in *Il10* ^−/−^ mice by promoting NK cell cytokine production and IL-12 thereby contributes indirectly to increased virus-specific CD4 T cell responses in *Il10* ^−/−^ mice.

Collectively, these data establish that IL-10 critically dampens the strength of the NK/DC crosstalk and thereby suppresses MCMV-specific CD4 T cell responses (Figure S11). Absence of IL-10 during MCMV infection unleashes a potent positive feedback loop between NK cells and DCs: NK cells produce more IFN-γ and TNF-α in MCMV-infected *Il10* ^−/−^ mice and, in combination with NKG2D and NCR-1 receptor engagement, promote DC activation which in turn facilitates priming of MCMV-specific CD4 T cells. Furthermore, DCs from MCMV-infected *Il10* ^−/−^ mice produce more IL-12, leading to exacerbated activation of NK cells and consequently virus-specific CD4 T cells. Unleashed NK/DC crosstalk in MCMV-infected *Il10* ^−/−^ mice therefore enables enhanced control of lytic viral replication via more potent activation of MCMV-specific CD4 T cell immunity, albeit at the expense of increased host pathology.

## Discussion

In order to regulate T cell mediated immunity during infections, in particular during chronic types of infection, IL-10 exerts pleiotropic direct and indirect suppressive effects towards different cell types of the innate and adaptive immune response [24]. The presence of an IL-10-mediated anti-inflammatory condition during early phases of an immune response can negatively affect the size and quality of the ensuing adaptive immune response. Such suppression of adaptive immunity may be pivotal in preventing harmful immune-mediated tissue damage for the host, but as a consequence is often associated with increased pathogen burden. For these reasons, viruses that cause chronic infections often exploit the IL-10 pathway in order to modulate the virus-host balance towards their own benefit. Here we establish that IL-10 markedly impairs priming of MCMV-specific CD4, but not CD8 T cell responses, by selectively attenuating maturation of CD8α^−^CD11b^+^CD11c^+^ myeloid DCs, which are crucial for the activation of MCMV-specific CD4 T cells. In contrast, IL-10 does not affect CD8α^+^ lymphoid organ-resident DCs, which are pivotal for the priming of MCMV-specific CD8 T cells [16]. This impaired MCMV-specific CD4 T cell priming in the presence of IL-10 results in prolonged MCMV replication together with limited TNF-α mediated immunopathology. Furthermore, we find that NK cells are a critical component promoting the maturation of myeloid DCs during MCMV infection of *Il10* ^−/−^ mice, since depletion of NK cells in *Il10* ^−/−^ reduces MCMV-specific CD4 T cell priming to levels observed in B6 mice. In the absence of IL-10, NK cells produce more IFN-γ and TNF-α, promoting, in combination with NKG2D and NCR-1 receptor engagement, the functional potency of MCMV-specific CD4 T cell priming by myeloid DCs. Moreover, in the absence of IL-10, DCs secrete more IL-12, which enhances activation and cytokine release of NK cells. Thus, the increased intensity of the NK/DC crosstalk plays a critical role for the massive priming of MCMV-specific CD4 T cell responses in the absence of IL-10. The downstream effect of an increased MCMV-specific CD4 T cell response in absence of IL-10 is accelerated control of lytic MCMV replication, which is, however, associated with more severe immunopathology.

Opposed to our results, immune-stimulatory effects of IL-10 on NK cells have also been reported, but mainly coming from *in vitro* studies [25]–[27]. In contrast to these *in vitro* data, a number of published *in vivo* studies [28], [29] also support a suppressive rather than activating effect of IL-10 on NK cells during an inflammatory response.

In our study, we used an MCMV mutant which lacks the *m157* gene, thereby abolishing the direct activation of NK cells via Ly49H. We aimed to assess whether strong NK cell activation through Ly49H would lead to dominance of NK cell cytotoxic activity towards DCs in the absence of IL-10, thus resulting in an impaired rather than an increased virus-specific CD4 T cell response in *Il10* ^−/−^ mice. However, this was not the case, since we corroborated our main findings by infecting mice with wt MCMV: *Il10* ^−/−^ mice harbored reduced virus loads 2 weeks post infection and virus-specific CD4 T cell priming was significantly increased in *Il10* ^−/−^ mice compared to B6 mice already at day 5.5 post infection (Figure S8). Furthermore, CD8α^−^CD11b^+^ myeloid DCs were markedly influenced in their activation phenotype in *Il10* ^−/−^ mice compared to B6 mice with higher expression levels of costimulatory markers (Figure S9B, C). Moreover, NK-like cells played a crucial role in promoting maturation of myeloid CD8α^−^CD11b^+^ DCs and CD4 T cell priming in the absence of IL-10, since depletion of NK-like cells abolished the enhanced activation phenotype of CD8α^−^CD11b^+^ DCs and CD4 T cell response in *Il10* ^−/−^ mice to the level of B6 control mice depleted of NK-like cells (Figure S9A). Furthermore, when we used a lower dose of viral inoculum, IL-10 suppression of NK/DC crosstalk and CMV-specific CD4 T cell priming was observed, indicating that the described effects are prominent even when the overall extent of inflammation is reduced (Figure S10).

Attenuation of DC maturation and function is a strategy actively exploited by herpes viruses to compromise the adaptive immune response of the host and thereby to prolong replication and consequently the chance for horizontal transmission. HCMV encodes an IL-10 homolog (cmv-IL10), which exerts suppressive effects on DC maturation, migration and cytokine secretion [5]. Here we document the importance of IL-10 in regulating the magnitude of this NK/DC crosstalk during MCMV infection with direct impact for the induction of adaptive antiviral immunity.

NK/DC cell to cell contact has been implied to be important in the cross-talk between these cell types. DCs are important targets of MCMV infection and the NKG2D receptor is considered to be involved in mutual interactions of NK cells and MCMV-infected DCs. However, the exact mechanism of this interaction still remains unknown. We observed that NK cells from *Il10^−/−^* mice show increased levels of the NKG2D and NCR-1 activating receptors as well as increased production of TNF-α and IFN-γ per cell level compared to B6 controls and we showed that all of those critically promote NK/DC crosstalk resulting in augmented virus-specific CD4 T cell priming. We have not considered a role of other NK cell receptors in this study, but it is conceivable that an overall threshold of signals coming from activatory and inhibitory NK cell receptors is shifted in the absence of IL-10.

Recently, in the context of HIV infection, it has been shown that IL-10 induction leads to changes in DC compartments and suppresses NK killing of immature DCs, thus promoting accumulation of poorly immunogenic APCs, which contribute to the immune dysfunction observed in HIV patients [30]. In the context of HCV infection, expression of the NKG2A inhibitory receptor on NK cells was shown to trigger production of the immunosuppressive cytokines IL-10 and TGF-β by NK cells, which suppressed the ability of DCs to prime Th1 polarized CD4 T cells [31].

The impact of NK cells on the regulation of virus-specific T cell responses during persistent infections has been evaluated in several studies. On one hand, NK cells promote MCMV-specific CD8 T cell responses by regulating the production of type I IFN by plasmacytoid DCs [32]. Moreover, strong activation of NK cells achieved by the use of recombinant Rae1γ MCMV virus induces protective CD8 T cell immunity and antibody responses [33]. In contrast, NK cells can also reduce the magnitude of T cell responses by directly lysing antigen-bearing immature DCs and thus curtailing T cell priming [21]. Ly49H-expressing NK cells lyse infected conventional DCs upon MCMV infection, thereby limiting virus-specific CD8 T cell priming [18]. IL-10 production by Ly49H expressing NK cells during uncontrolled MCMV infection of *Prf1* ^−/−^ mice has been suggested to play an important role in limiting CD8 T cell responses and immunopathology [34]. Moreover, very recent reports have shown that NK cells can have a direct cytotoxic effect towards activated CD4 and CD8 T cells during chronic LCMV Clone 13 infection with an impact on virus-induced immunopathology [35], [36]. Thus, there seems to be a controversy with two opposing effects of NK cells in either promoting or suppressing virus-specific T cell responses during viral infection. It has been proposed that the ratio of NK cells and DCs could direct the role of NK cells either towards promotion or restriction of virus-specific T cell responses *in vivo* [18], [37]. Specifically, the presence of high numbers of NK cells vs. DCs during an infection would lead to predominance of NK cell cytotoxicity towards immature DCs, suppressing the priming of virus-specific T cells. In contrast, low numbers of NK cells vs. DCs would lead to NK/DC interactions that stimulate priming of the virus-specific T cell response. Taking into account that IL-10 reduces activation-induced NK cell death and accumulation of cytotoxic NK cells during the acute phase of MCMV infection [19], one could argue that early induction of IL-10 during MCMV infection may lead to high NK/DC ratios, a scenario in which NK cells suppress T cell priming by lysing DCs. Our data are in line with such a hypothesis, since we demonstrate that immunogenic NK/DC crosstalk is attenuated and overridden by IL-10 with direct consequences for the induction of protective CD4 T cell immunity.

Upon infection, IL-10 can be secreted by many different cell types [3]. T and B cells, DCs, macrophages and NK cells produce IL-10 during various chronic viral infections [24]. During MCMV infection, IL-10 secretion by CD4 T cells and B cells, inflammatory macrophages and DCs has been reported [7], [19], [38]. Here we confirmed that MCMV infection induces IL-10 production, assessed by IL-10 reporter activity, by various cell types, including myeloid DCs, macrophages, NK cells, CD4 T cells and B cells. Thus, the relative contribution of each IL-10 producing cell type to the overall *in vivo* effects of this immunosuppressive cytokine is likely very complex and it might also differ depending on the tissue and the time of infection. We identified CD11c^+^ cells and to some extent macrophages, but not CD4 T cells, to be important *in vivo* sources of IL-10 during acute MCMV infection using mixed bone marrow chimeras in which specific cell types were IL-10 deficient. Although CD4 T cells showed prominent IL-10 reporter activity during MCMV infection, CD4 T cell derived IL-10 was dispensable for constraining MCMV-specific CD4 T cell responses and virus control. This discrepancy either indicates that IL-10 reporter activity does not accurately reflect IL-10 protein production *in vivo*, perhaps due to posttranscriptional regulation as shown for NK cells [39], or that CD4 T cell-derived IL-10 does not contribute to suppress MCMV-specific CD4 T cell responses because of potential anatomical or kinetic constraints of the IL-10 source which are not met by CD4 T cells. Also, it will be of interest to assess the *in vivo* role of NK cells as an early source of IL-10 during MCMV infection in future studies.

A recent report addressed the impact of IL-10 on MCMV-specific CD8 T cell responses during the latent phase of MCMV infection [13]; although IL-10 did not alter early priming of MCMV-specific CD8 T cells, it limited CD8 T cell memory inflation. It is conceivable that the increased memory CD8 T cell inflation in the absence of IL-10 is a direct consequence of the greatly increased MCMV-specific CD4 T cell response, as CD4 T cells support memory CD8 T cell inflation [40]. In line with this hypothesis, CD8 T cells specific for the IE-3 epitope, previously shown to be highly dependent on CD4 T cell help [40], [41], were profoundly increased in *Il10* ^−/−^ mice.

In conclusion, we identified an important role of IL-10 during early MCMV infection and its impact for priming of MCMV-specific CD4 T cell responses. By suppressing the innate-adaptive immune cell crosstalk and, in particular, regulating the strength of NK/DC interactions, IL-10 specifically limits the activation of virus-specific CD4 T cells, which in turn leads to reduction of host tissue damage but promotes virus persistence in the long run (Fig. S11). These two opposing effects of IL-10 in regulating the virus-host balance during primary CMV infection should be taken into account when considering therapeutic properties of this immunosuppressive cytokine.

## Materials and Methods

### Ethics statement

This study was conducted in accordance to the guidelines of the animal experimentation law (SR 455.163; TVV) of the Swiss Federal Government. The protocol was approved by Cantonal Veterinary Office of the canton Zurich, Switzerland (Permit number 145/2008, 109/2011).

### Mice, viruses, peptides and *in vivo* antibody treatment

C57BL/6J, *Il10* ^−/−^, *Il10rβ* ^−/−^ mice were bred in the local animal facility under specific pathogen free conditions. *Il10* GFP knock-in Tiger mice were generated and provided by Dr. R. A. Flavell (Yale University, USA). DC-*Il10r* ^−/−^ [22] and CD11c-DTR/GFP mice [42] were previously described and LysMCre/iDTR mice were kindly provided by Dr. T. Buch (Technical University, Munich, Germany). BAC-derived MCMV MW97.01 (WT MCMV in the text) and recombinant MCMV *Δm157* were previously described [17] and were propagated on C57BL/6 embryonic fibroblasts (MEFs). Viral titers were determined by standard virus plaque assay [43]. Mice were infected intravenously (i.v.) with 5×10^6^ plaque forming units (PFU) of MCMV. The MCMV derived m14_aa136–150_, M25_aa411–425_, M25_aa721–735_, M112_aa36–50_, m142_aa26–40_ m139_560–574_, M45_aa985–993_, M38_aa316–323_ peptides were purchased from NeoMPS (Strasbourg, France). CD8 and CD4 T cells were depleted *in vivo* with 0.2 mg of purified anti-mouse CD8 and anti-mouse CD4 monoclonal antibodies (YTS 169.4 respectively YTS 191.1). Mice were injected i.p. 3 and 1 days before infection and then weekly. NK-like cells were depleted with 0.5 mg of anti-NK.1.1 monoclonal antibody (PK136, BioXCell). Mice were injected i.p. at the day 1 before infection and every second day post infection. TNF-α, IFN-γ and IL-12 were neutralized with 0.5 mg of anti-TNF-α (XT3.11, BioXCell), anti-IFN-γ (XMG1.2, BioXCell) and anti-IL-12 (C17.8, BioXCell) antibodies and NKG2D was neutralized with 0.5 mg of anti-NKG2D (HMG2D, BioXCell) antibody. Mice were injected i.p. at day of infection and on days 3 and 4 post infection except for anti-IFN-γ where mice were injected at day 3 and 4 post infection.

### Antibodies and tetramers

APC-conjugated peptide-MHC class I tetramers were generated as described (Altman JD, 1996). The following fluorochrome-conjugated antibodies were obtained either from Biolegend (Lucerna-Chem AG, Luzern, Switzerland) or from BD Biosciences (Allschwil, Switzerland): anti-CD4, anti-CD8, anti-NK1.1, anti-CD3ε, anti-TCRVα11.1, anti-CD11c, anti-CD11b, anti-B220, anti-I-A^b^, anti-Ly6C, anti-TNF-α, anti-IFN-γ, anti-CD45.1, anti-CD40, anti-CD86, anti-CD80 and anti-NKG2D.

### Stimulation of lymphocytes, cell surface and intracellular stainings and flow cytometry

Lymphocytes were isolated from spleen, lung, liver, lymph nodes and salivary gland as previously described [17], [44]. For intracellular cytokine stainings, CD4 T cells were first stimulated with 3 µg/ml of CD4 peptide pool (m14_aa136–150_, M25_aa411–425_, M25_aa721–735_, M112_aa36–50_, m142_aa26–40_ m139_560–574_) and CD8 T cells were restimulated with 1 µg/ml of M45_aa985–993_, M38_aa316–323_ peptides in the presence of 10 µg/ml brefeldin A (Sigma Aldrich) at 37°C for 6 hours. Cells were stained for cell surface markers with directly conjugated antibodies and incubated for 20 min at 4°C or at 37°C when using tetramers. For intracellular cytokine stainings cells were then fixed and permeabilized using Fix/Perm solution (FACSLyse diluted to 2X concentration and 0.05% Tween 20) for 10 min at room temperature. Cells were then washed and stained with directly labeled anti-IFN-γ and anti-TNF-α antibodies for 20 min at 4C. Multiparameter FACS analysis was performed on a LSRII flow cytometer (BD Bioscience) using FACS DIVA software (BD Bioscience). Data were analysed using FlowJo software (Treestar).

### CD4 T cell proliferation assay

M25-II cells were isolated by MACS from splenocytes of naive M25-II transgenic mice by positive selection with anti-CD4 microbeads (Miltenyi Biotech). Cells were labeled with 0.5 µM CFSE (Invitrogen) for 8 min at 37°C. CD11c^+^ cells were isolated by MACS from spleens of B6 and *Il10* ^−/−^ mice by positive selection with anti-CD11c microbeads (Miltenyi). 1×10^5^ CD11c^+^ cells of infected B6 and *Il10* ^−/−^ mice were loaded with 10^−8^ M of M25 peptide and co-cultured with 6×10^4^ CFSE labeled M25-II cells. After 3 days of incubation, cells were stained with anti-CD4 and anti-CD45.1 antibodies and CFSE dilution was measured by flow cytometry.

### NK/DC/CD4 T cell proliferation assay

M25-II cells were isolated and CFSE labeled as described above. Naive CD11c^+^ cells were isolated from the spleens of B6 and *Il10r* ^−/−^ mice as described above. NK cells were isolated from the spleens of infected B6 and *Il10* ^−/−^ mice at day 3.5 post infection as previously described [45]. Briefly, splenocytes were first negatively depleted of T cells using FITC-conjugated anti-CD3ε and anti-FITC microbeads (Miltenyi). NK cells were then positively selected by staining the T cell-depleted fraction with anti-DX5 beads (Miltenyi). 2×10^5^ of purified NK cells were co-cultured with 6×10^4^ CFSE labeled M25-II cells and 1×10^4^ CD11c^+^ cells in 96-U bottom plates and treated with or without 50 µg/ml anti-IFN-γ, 50 µg/ml anti-TNF-α or 3 µg/ml anti-NKG2D antibodies (all purchased from BioXcell) and 5 µg/ml of purified NCR-1 monoclonal antibody (provided by Prof. S. Jonjic). After 3 days of incubation, cells were stained with anti-CD4 and anti-CD45.1 antibodies and CFSE dilution was measured by flow cytometry.

## Supporting Information

Figure S1 IL-10 does not influence MCMV-specific CD8 T cell responses during acute MCMV infection.
(DOC)

Figure S2 IL-10 does not act directly on CD4 T cells during acute MCMV infection.
(DOC)

Figure S3 IL-10 does not influence CD8α^+^ DC phenotype during acute MCMV infection.
(DOC)

Figure S4 Generation of MHC class II-restricted TCR transgenic mice with specificity for the CD4 T cell epitope of the MCMV protein M25.
(DOC)

Figure S5 CD11c^+^ cells and macrophages/neutrophils are a relevant source of IL-10 upon MCMV infection.
(DOC)

Figure S6 Depletion of NK1.1+ cells, neutralization of IFN-γ, TNF-α, IL-12 and blocking of NKG2D enhances the virus titers in B6 and *Il10^−/−^* mice.
(DOC)

Figure S7 Dendritic cells are responsive to NK cell derived factors induced by MCMV infection.
(DOC)

Figure S8 Increased CD4 T cell response and decreased lytic viral replication in *Il10* ^−/−^ mice upon acute infection with wt MCMV.
(DOC)

Figure S9 NK-like cells are responsible for increased CD4 T cell responses and promote DC maturation in *Il10* ^−/−^ mice during acute infection with wt MCMV.
(DOC)

Figure S10 IL-10 suppresses NK/DC crosstalk and MCMV-specific CD4 T cell priming at low dose of MCMV inoculums.
(DOC)

Figure S11 IL-10 dampens DC/NK cross-talk during MCMV infection.
(DOC)

Text S1 Materials and methods for supplementary figures.
(DOC)
