# Supplementary material for: IL-10 Suppression of NK/DC Crosstalk Leads to Poor Priming of MCMV-Specific CD4 T Cells and Prolonged MCMV Persistence
Source: PLoS Pathog. 2012 Aug 2;8(8):e1002846. doi: 10.1371/journal.ppat.1002846 (PMC3410900; doi:10.1371/journal.ppat.1002846)
Supplement: Figure S1 — IL-10 does not influence MCMV-specific CD8 T cell responses during acute MCMV infection. (DOC) [file ppat.1002846.s001.doc]

**
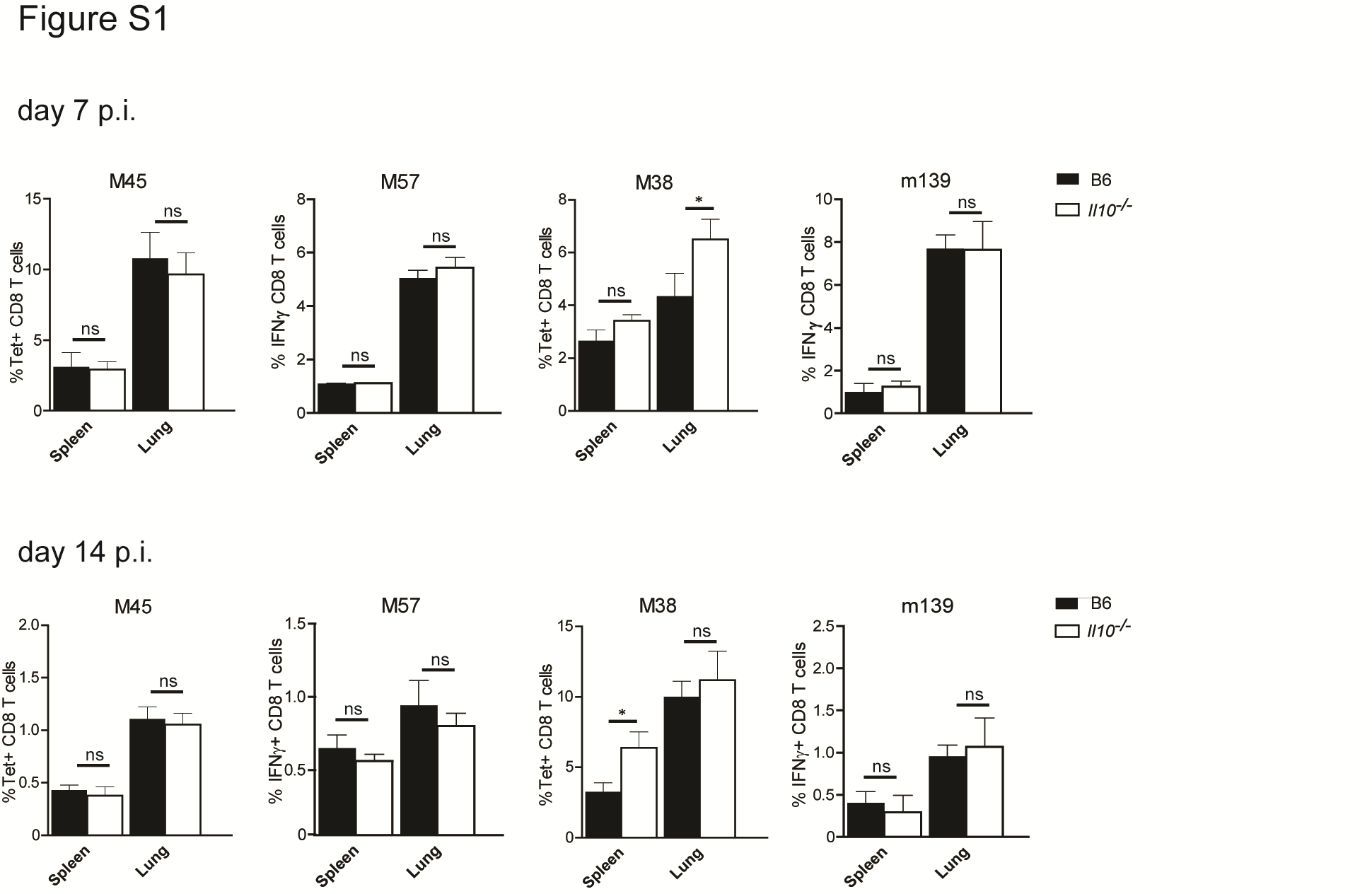
**

**Figure S1 IL-10 does not influence MCMV-specific CD8 T cell responses during acute MCMV infection**

B6 and *Il10*^-/-^ mice were infected with 5x10^6^ PFU *Δm157* MCMV. Lymphocytes were isolated from spleen and lungs on day 7 (upper panel) and 14 p.i. (lower panel). M38- and M45-specific CD8 T cell responses were measured by tetramer staining, whereas M57- and m139- CD8 T cell responses were measured by intracellular staining for IFN-γ upon *ex vivo* restimulation with appropriate peptides (M57 _816–824_; and m139 _419–426_). The percentages of M45- and M38-tetramer positive cells as well as M57- and m139-IFN-γ producing cells among total CD8 T cells are shown.
