# Supplementary material for: IL-10 Suppression of NK/DC Crosstalk Leads to Poor Priming of MCMV-Specific CD4 T Cells and Prolonged MCMV Persistence
Source: PLoS Pathog. 2012 Aug 2;8(8):e1002846. doi: 10.1371/journal.ppat.1002846 (PMC3410900; doi:10.1371/journal.ppat.1002846)
Supplement: Figure S2 — IL-10 does not act directly on CD4 T cells during acute MCMV infection. (DOC) [file ppat.1002846.s002.doc]

**
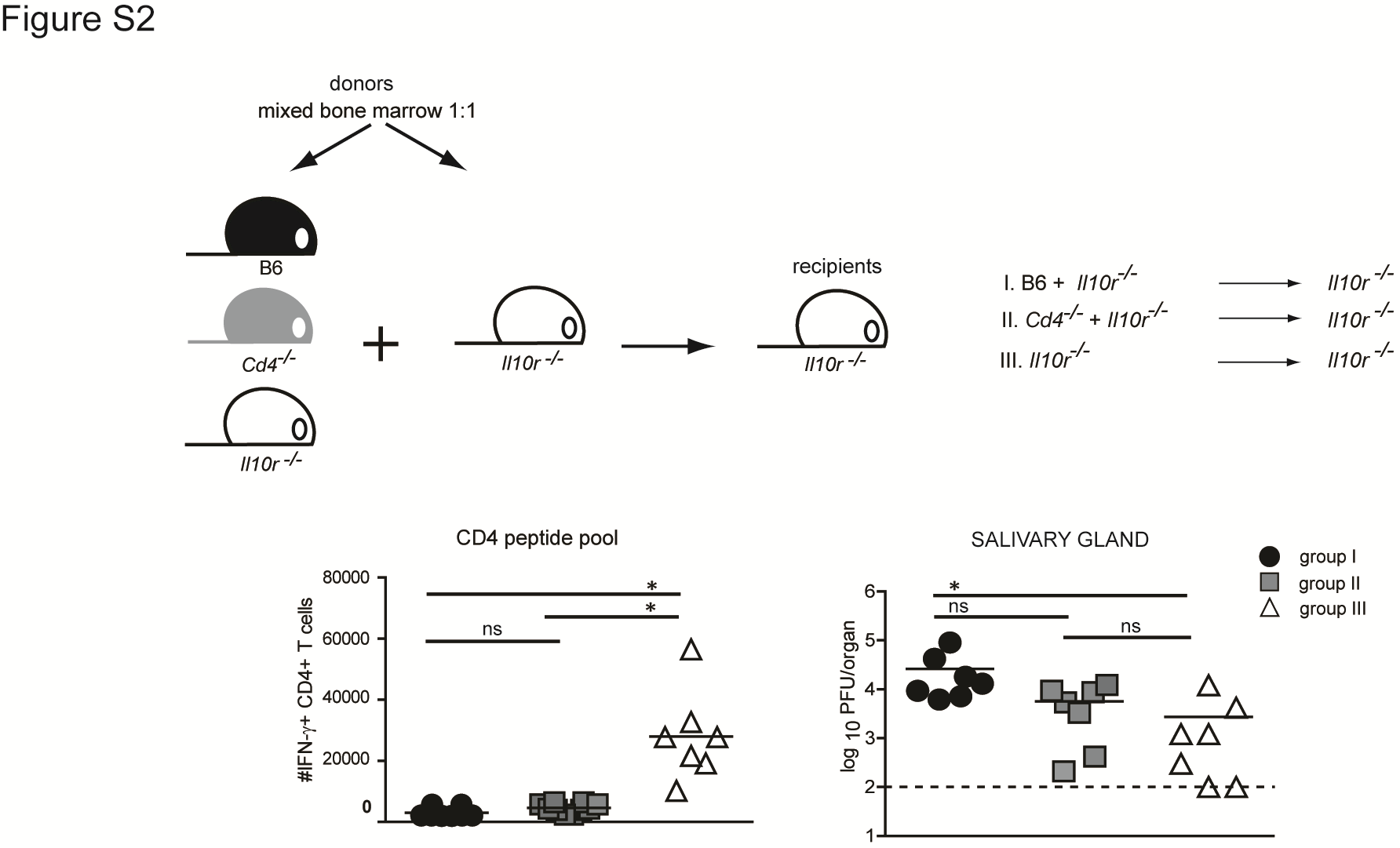
**

**Figure S2 IL-10 does not act directly on CD4 T cells during acute MCMV infection**

Mixed bone marrow chimeras harboring CD4 T cells capable (B6 + *Il10r*^-/-^ → *Il10r*^-/-^) or incapable to sense IL-10 (*Cd4*^-/-^ + *Il10r*^-/-^ → Il10r^-/-^) were generated. As control, bone marrow chimeras with complete absence of IL-10R were generated (*Il10r*^-/-^ → *Il10r*^-/-^). After reconstitution, mice were infected with 5x10^6^ PFU *Δm157* MCMV. Lung lymphocytes were isolated at day 14 p.i. and *ex vivo* restimulated with the CD4 peptide pool (M14, m18, M25, M112, m139 and m142 peptides). Numbers of IFN-γ^+^ TNF-α^+^ peptide-specific CD4 T cells from all three groups of chimeras are shown (left graph). Virus titers in salivary glands on day 14 p.i. are shown (right graph). Each symbol represents one individual mouse, horizontal line indicates the mean (n=3-4), dashed line indicates the detection limit. Statistical analysis was performed by 2-tailed unpaired student's t-test (* p<0.05). The data are representative of 2 pooled experiments.
