# Supplementary material for: IL-10 Suppression of NK/DC Crosstalk Leads to Poor Priming of MCMV-Specific CD4 T Cells and Prolonged MCMV Persistence
Source: PLoS Pathog. 2012 Aug 2;8(8):e1002846. doi: 10.1371/journal.ppat.1002846 (PMC3410900; doi:10.1371/journal.ppat.1002846)
Supplement: Figure S3 — IL-10 does not influence CD8α+ DC phenotype during acute MCMV infection. (DOC) [file ppat.1002846.s003.doc]

**
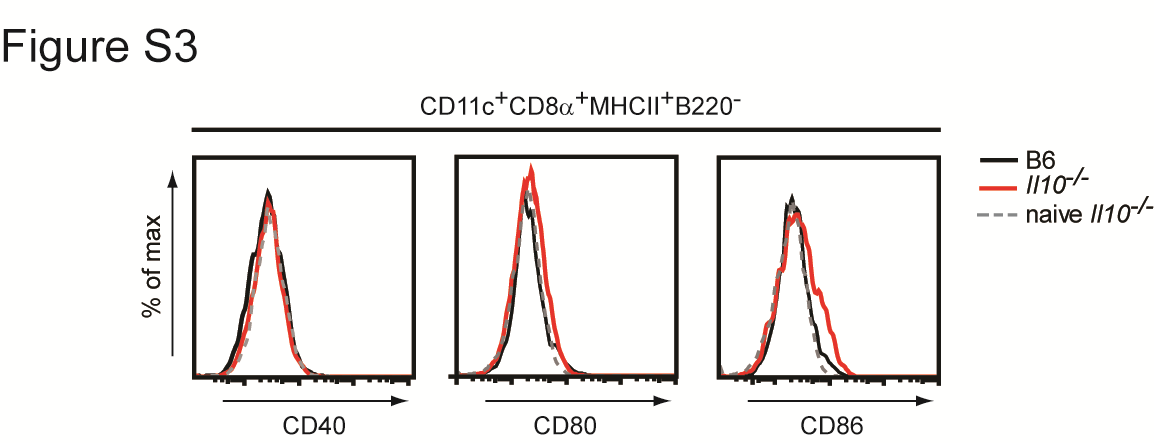
**

**Figure S3 IL-10 does not influence CD8α^+^ DC phenotype during acute MCMV infection**

B6 and *Il10*^-/-^ mice were infected with 5x10^6^ PFU *Δm157* MCMV. Splenocytes from infected mice were isolated at day 5.5 post infection. Representative FACS plots showing expression levels of CD40, CD80 and CD86 costimulatory molecules. Plots are gated on CD11c^+^CD8α^+^MHCII^+^B220^-^ DCs.
