# Supplementary material for: IL-10 Suppression of NK/DC Crosstalk Leads to Poor Priming of MCMV-Specific CD4 T Cells and Prolonged MCMV Persistence
Source: PLoS Pathog. 2012 Aug 2;8(8):e1002846. doi: 10.1371/journal.ppat.1002846 (PMC3410900; doi:10.1371/journal.ppat.1002846)
Supplement: Figure S4 — Generation of MHC class II-restricted TCR transgenic mice with specificity for the CD4 T cell epitope of the MCMV protein M25. (DOC) [file ppat.1002846.s004.doc]

**
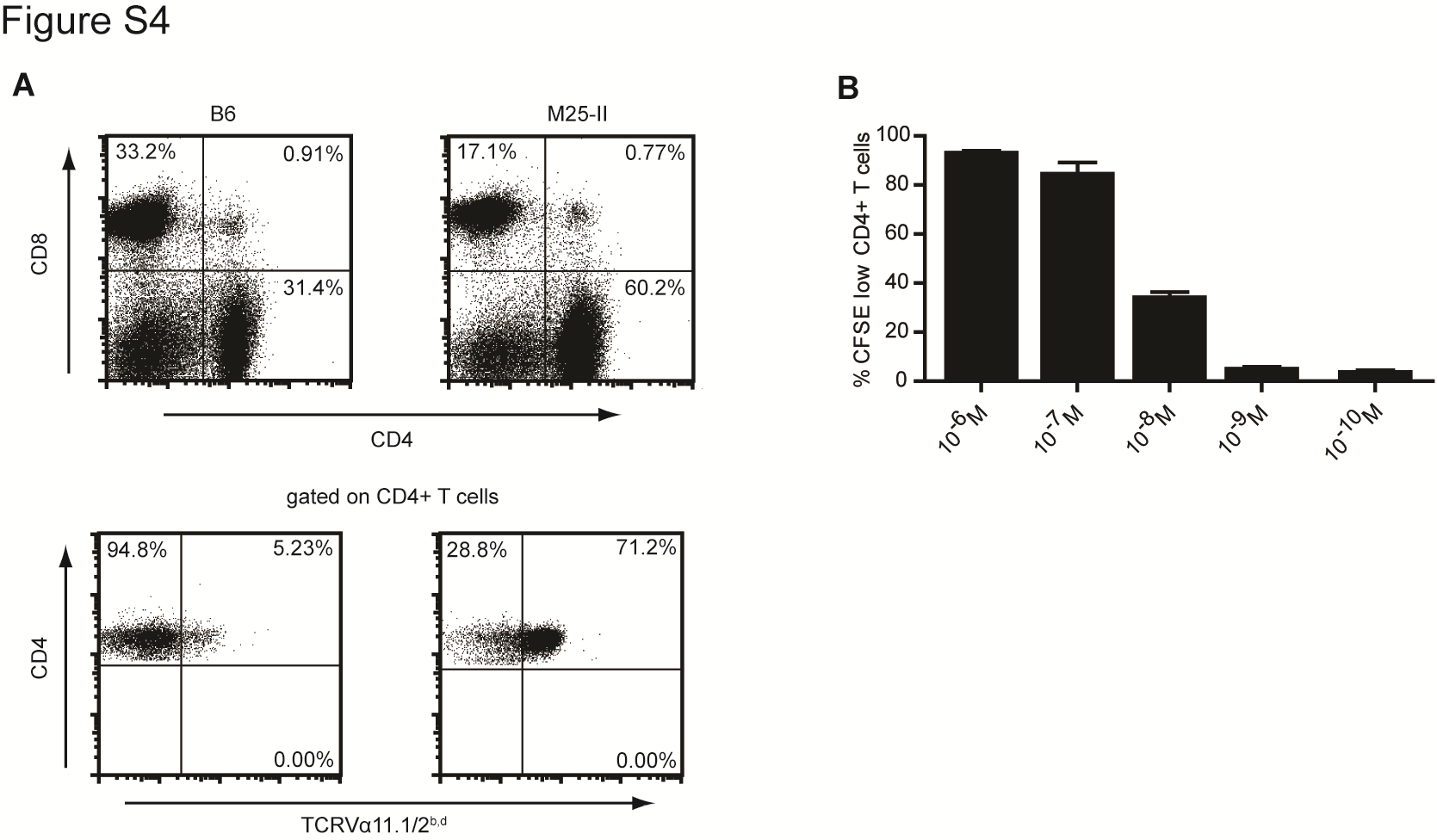
**

**Figure S4 Generation of MHC class II-restricted TCR transgenic mice with specificity for the CD4 T cell epitope of the MCMV protein M25**

A) Lymph nodes from B6 and M25-II transgenic mice were stained with anti-CD4, anti-CD8 and anti-TCRVα11.1/2^b,d^ antibodies. Representative plots with percentages of CD4 and CD8 T cells (upper row) and TCRVα11.1/2^b,d+^ CD4 T cells (lower row) are shown. Data are representative of 2 independent experiments. B) Naive M25-II CD4 T cells were CFSE labeled and incubated with naive B6 splenocytes loaded with the indicated amounts of M25 peptide for 3 days. Percentages of CFSE low CD4 T cells are shown (n=3, error bars indicate standard deviation, data are representative of 2 independent experiments). Statistical analysis was performed by 2-tailed unpaired student's t-test (* p<0.05, ** p<0.01, *** p<0.001).
