# Supplementary material for: IL-10 Suppression of NK/DC Crosstalk Leads to Poor Priming of MCMV-Specific CD4 T Cells and Prolonged MCMV Persistence
Source: PLoS Pathog. 2012 Aug 2;8(8):e1002846. doi: 10.1371/journal.ppat.1002846 (PMC3410900; doi:10.1371/journal.ppat.1002846)
Supplement: Figure S5 — CD11c+ cells and macrophages/neutrophils are a relevant source of IL-10 upon MCMV infection. (DOC) [file ppat.1002846.s005.doc]

**
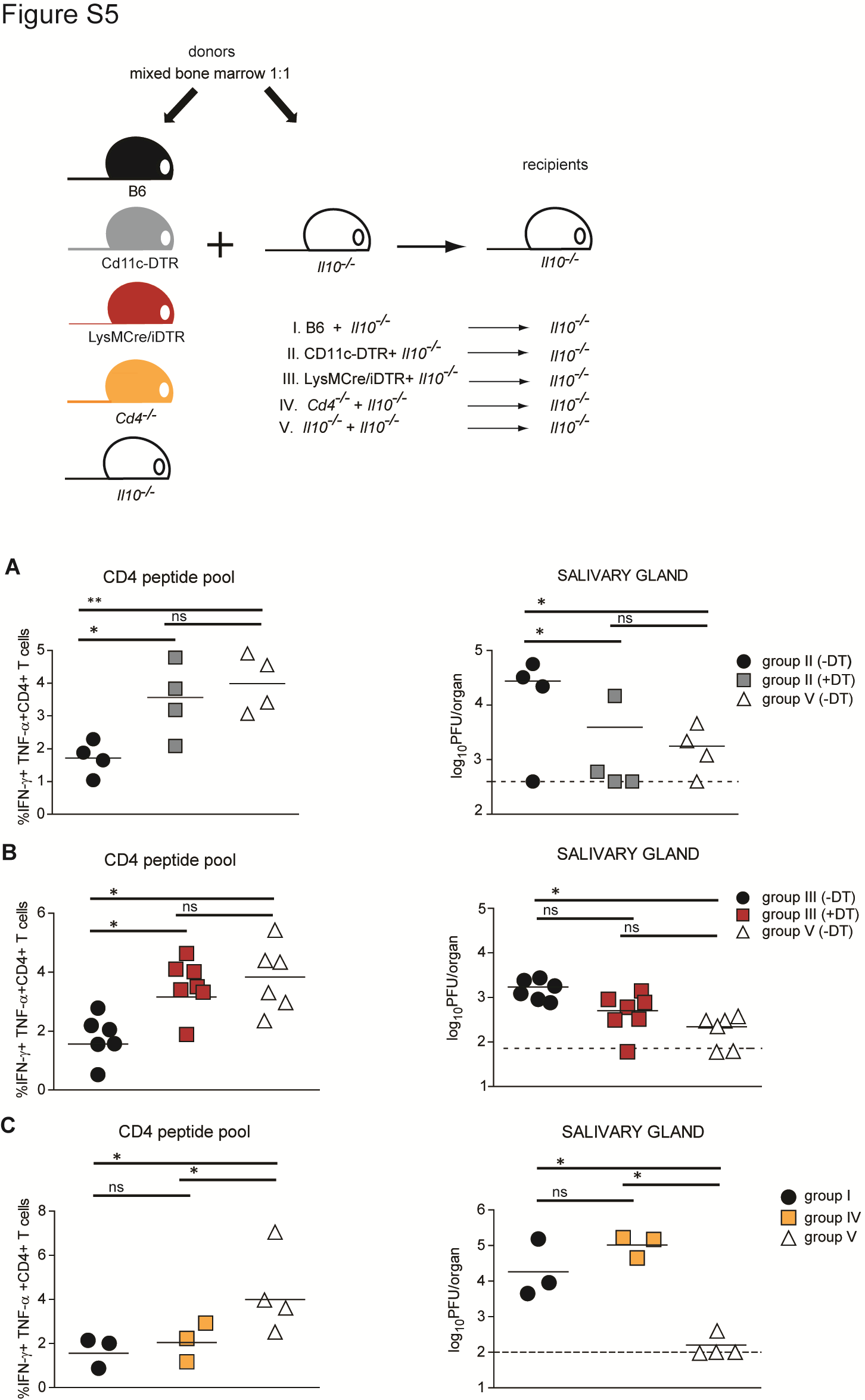
**

**Figure S5 CD11c^+^ cells and macrophages/neutrophils are a relevant source of IL-10 upon MCMV infection**

Mixed bone marrow chimeras harboring CD11c^+^ cells (A), macrophages or neutrophils (B) and CD4 T cells (C) capable or incapable of producing IL-10 were generated. (A) Chimeras without DT administration harbor CD11c^+^ cells able to produce IL-10, chimeras with DT administration harbor CD11c^+^ cells incapable of producing IL-10. (B) Chimeras without DT administration harbor macrophages and neutrophils able to produce IL-10, chimeras with DT administration harbor macrophages and neutrophils incapable of producing IL-10. (C) Group I chimeras harbor CD4 T cells capable of producing IL-10, group IV chimeras harbor CD4 T cells incapable of producing IL-10. B6, *Il10*^-/-^, CD11c-DTR (A), LysMCre/iDTR (B) and *Cd4*^-/-^ (C) mice were used as bone marrow donors and *Il10*^-/-^ mice were used as a recipients. After reconstitution, mice were infected with *Δm157* MCMV and treated with DT or PBS (A, B). Lung lymphocytes were isolated and *ex vivo* restimulated with the CD4 peptide pool (M14, m18, M25, M112, m139 and m142 peptides). Percentages of IFN-γ^+^ TNF-α^+^ peptide- specific CD4 T cells are shown (left column). Virus titers in salivary glands on day 14 post infection are shown (right column). Each symbol represents one individual mouse, horizontal line indicates the mean (n=3-4), dashed line indicates the detection limit. Data are representative of 2 independent experiments (A and C) and 2 pooled experiments (B). Statistical analysis was performed by 2-tailed unpaired student's t-test (* p<0.05, ** p<0.01, *** p<0.001).
