# Supplementary material for: IL-10 Suppression of NK/DC Crosstalk Leads to Poor Priming of MCMV-Specific CD4 T Cells and Prolonged MCMV Persistence
Source: PLoS Pathog. 2012 Aug 2;8(8):e1002846. doi: 10.1371/journal.ppat.1002846 (PMC3410900; doi:10.1371/journal.ppat.1002846)
Supplement: Figure S6 — Depletion of NK1.1+ cells, neutralization of IFN-γ, TNF-α, IL-12 and blocking of NKG2D enhances the virus titers in B6 and Il10−/− mice. (DOC) [file ppat.1002846.s006.doc]

**
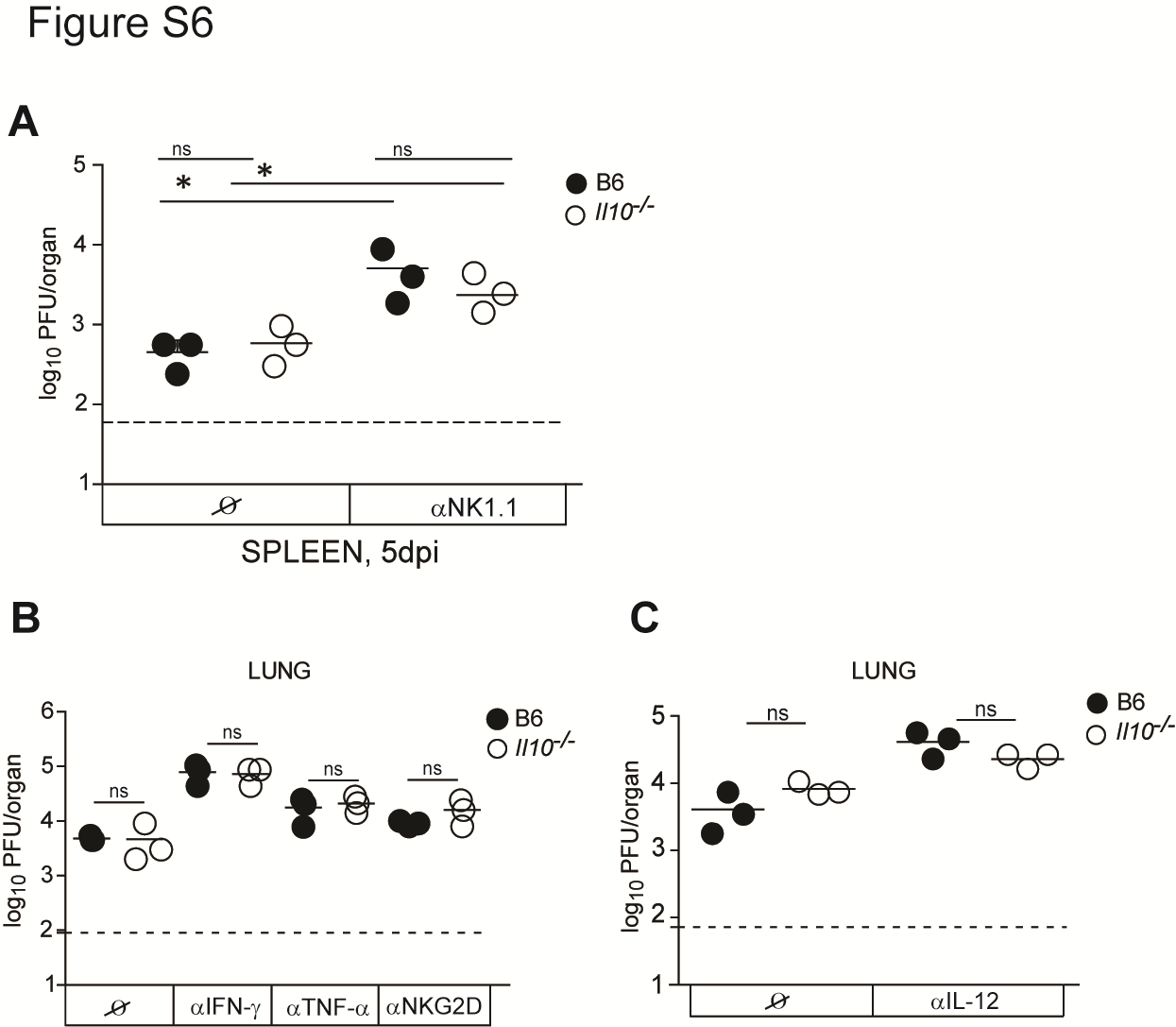
**

**Figure S6 Depletion of NK1.1+ cells, neutralization of IFN-γ, TNF-α, IL-12 and blocking of NKG2D enhances the virus titers in B6 and *Il10^-/-^* mice**

B6 and *Il10*^-/-^ mice were infected with 5x10^6^ PFU *Δm157* MCMV and treated with αNK1.1 depleting antibody at days 0, 2, 4 p.i, with αIFN-γ antibody at days 3, 4 p.i. and with αTNF-α and αNKG2D at days 0, 3, 4 p.i. A, B, C). Virus titers at day 5.5 p.i. were determined in lungs. Each symbol represents one individual mouse, horizontal line indicates the mean, dashed line indicates the detection limit (n=3). Data are representative of 3 independent experiments. Statistical analysis was performed by 2-tailed unpaired student's t-test (** p<0.01, *** p<0.001, n.d. = not detected).
