# Supplementary material for: IL-10 Suppression of NK/DC Crosstalk Leads to Poor Priming of MCMV-Specific CD4 T Cells and Prolonged MCMV Persistence
Source: PLoS Pathog. 2012 Aug 2;8(8):e1002846. doi: 10.1371/journal.ppat.1002846 (PMC3410900; doi:10.1371/journal.ppat.1002846)
Supplement: Figure S7 — Dendritic cells are responsive to NK cell derived factors induced by MCMV infection. (DOC) [file ppat.1002846.s007.doc]

**
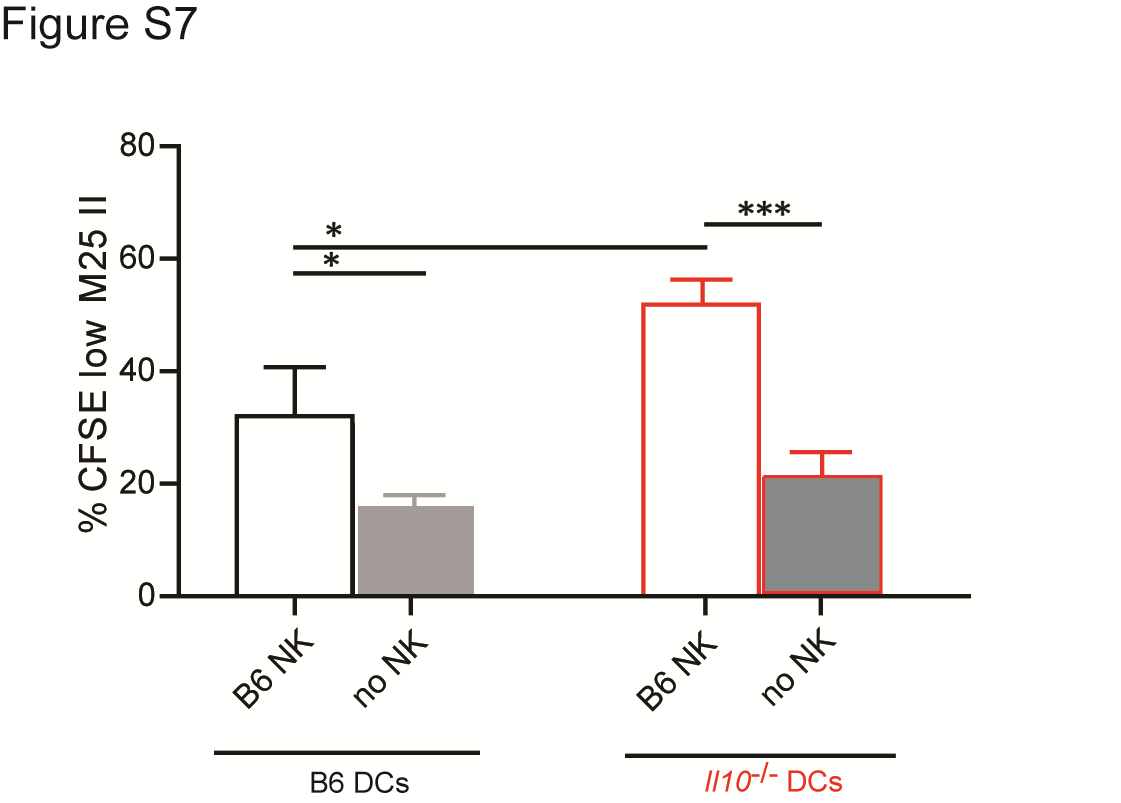
**

**Figure S7 Dendritic cells are responsive to NK cell derived factors induced by MCMV infection**

B6 were infected with 5x10^6^ PFU *Δm157* MCMV and NK cells were isolated from the spleens of infected B6 mice at day 3.5 post infection as previously described. M25-II cells were isolated by MACS from splenocytes of naive M25-II transgenic mice and labeled with CFSE. CD11c^+^ cells were enriched by MACS purification from spleens of B6 and *Il10*^-/-^ , loaded with M25 peptide and incubated with CFSE-labeled M25-II transgenic cells with or without (indicated as “no NK”) addition of NK cells. The frequencies of CFSE^low^ M25 II cells were assessed on day 3 of respective cultures.
