# Supplementary material for: IL-10 Suppression of NK/DC Crosstalk Leads to Poor Priming of MCMV-Specific CD4 T Cells and Prolonged MCMV Persistence
Source: PLoS Pathog. 2012 Aug 2;8(8):e1002846. doi: 10.1371/journal.ppat.1002846 (PMC3410900; doi:10.1371/journal.ppat.1002846)
Supplement: Figure S8 — Increased CD4 T cell response and decreased lytic viral replication in Il10 −/− mice upon acute infection with wt MCMV. (DOC) [file ppat.1002846.s008.doc]

**
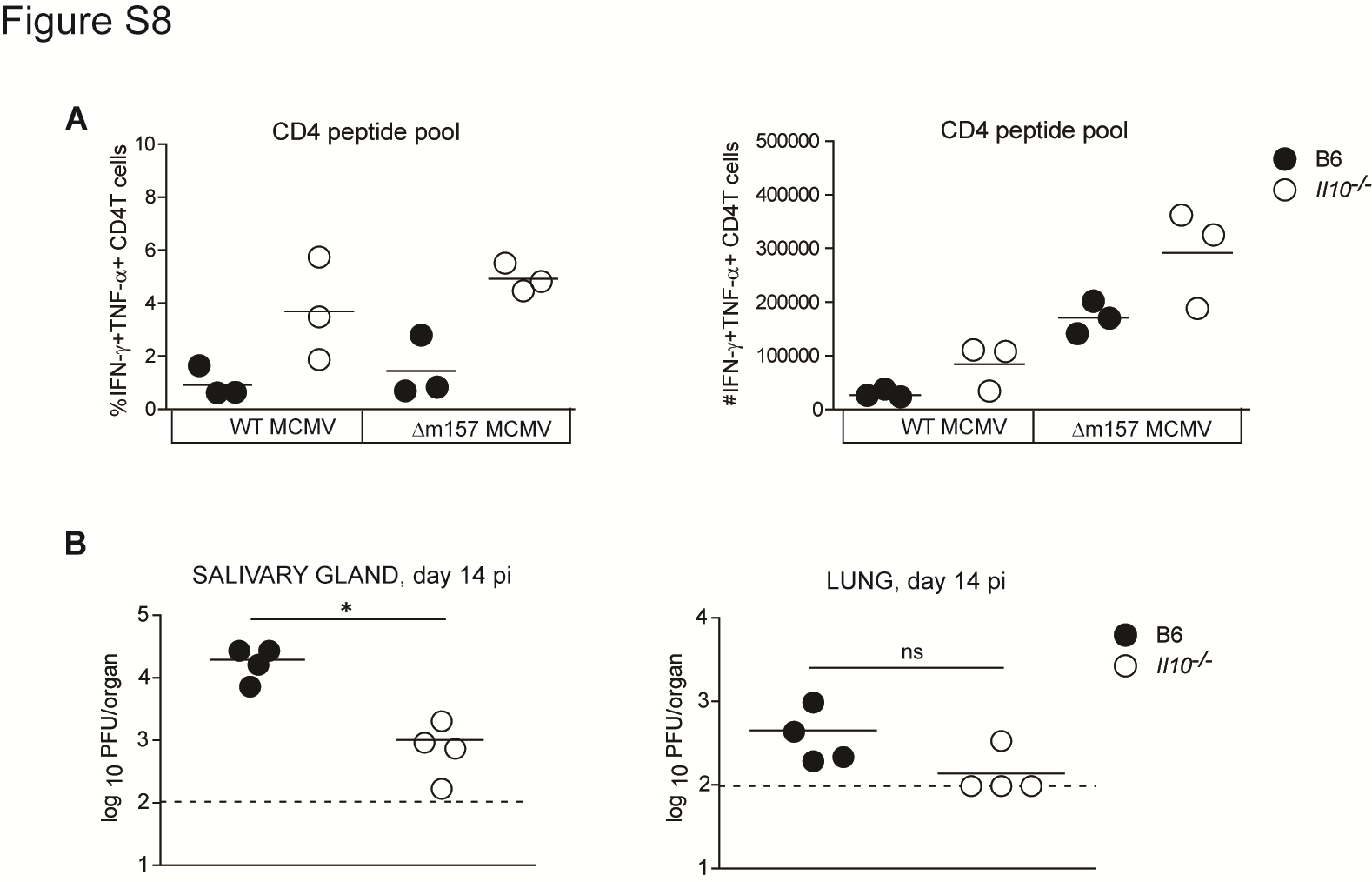
**

**Figure S8 Increased CD4 T cell response and decreased lytic viral replication in *Il10*^-/-^ mice upon acute infection with wt MCMV**

A) B6 and *Il10*^-/-^ mice were infected with 5x10^6^ PFU WT MCMV or 5x10^6^ PFU *Δm157* MCMV. Lung lymphocytes were isolated at day 14 p.i. and *ex vivo* restimulated with the CD4 peptide pool (M14, m18, M25, M112, m139 and m142 peptides). Percentages and total numbers of IFN-γ^+^ TNF-α^+^ peptide specific CD4 T cells from B6 and *Il10*^-/-^ mice are shown (n=3, data are representative 2 independent experiments). B) B6 and *Il10*^-/-^ mice were infected with 5x10^6^ PFU WT MCMV. Virus titers in salivary glands and lungs of B6 and *Il10*^-/-^ mice at day 14 p.i. are shown (n=3, each symbol represents one individual mouse, horizontal line indicates the mean, dashed line indicates the detection limit). Data are representative of 2 independent experiments. Statistical analysis was performed by 2-tailed unpaired student's t-test (* p<0.05, ** p<0.01, *** p<0.001).
