# Supplementary material for: IL-10 Suppression of NK/DC Crosstalk Leads to Poor Priming of MCMV-Specific CD4 T Cells and Prolonged MCMV Persistence
Source: PLoS Pathog. 2012 Aug 2;8(8):e1002846. doi: 10.1371/journal.ppat.1002846 (PMC3410900; doi:10.1371/journal.ppat.1002846)
Supplement: Figure S10 — IL-10 suppresses NK/DC crosstalk and MCMV-specific CD4 T cell priming at low dose of MCMV inoculums. (DOC) [file ppat.1002846.s010.doc]

**
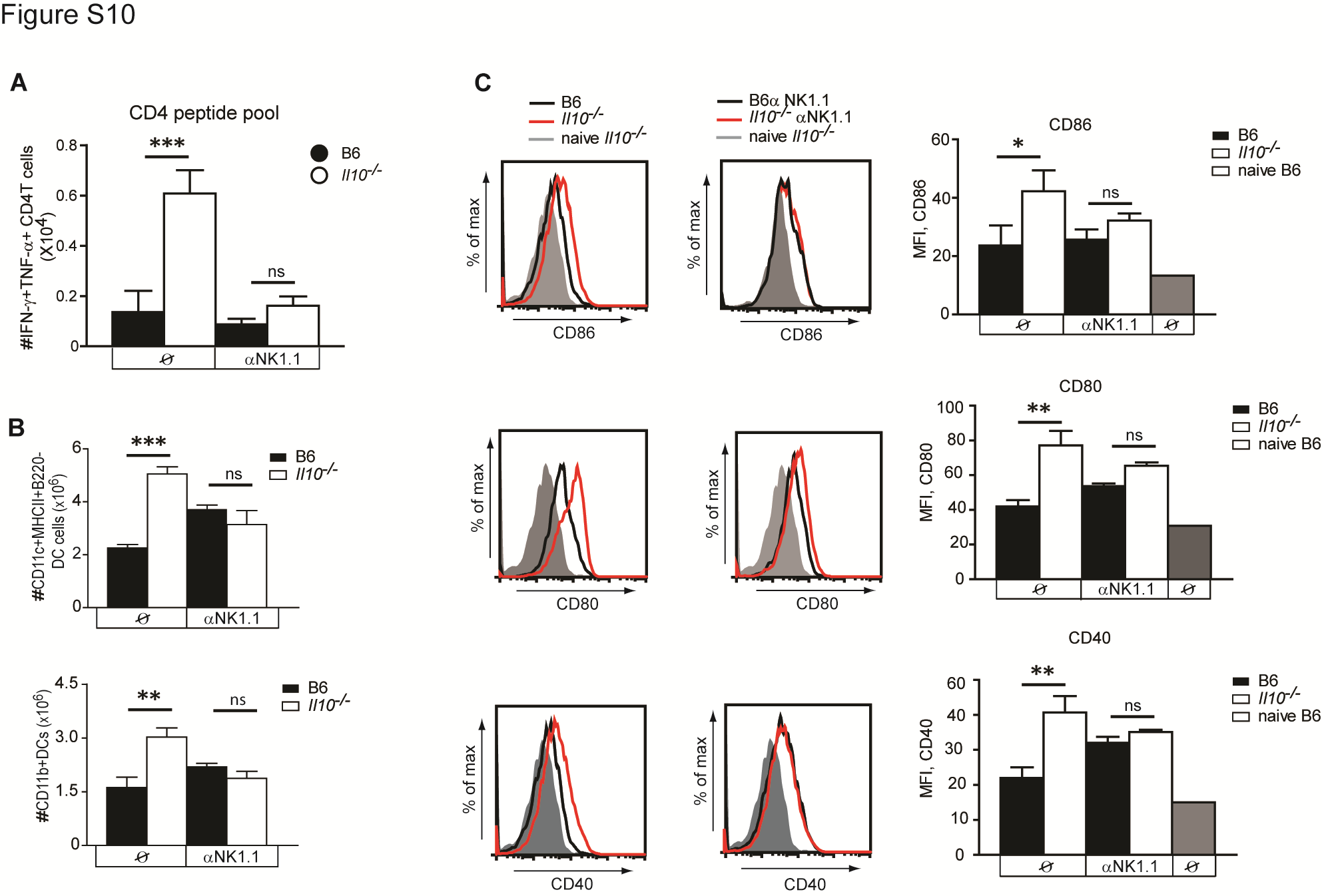
**

**Figure S10 IL-10 suppresses NK/DC crosstalk and MCMV-specific CD4 T cell priming at low dose of MCMV inoculum**

A) B6 and *Il10*^-/-^ mice were infected with 10^4^ PFU *Δ*m157 MCMV and either mock treated or depleted of NK-like cells using αNK1.1 (PK136) antibody. Lung lymphocytes were isolated at day 5.5 post infection and *ex vivo* restimulated with the CD4 peptide pool (M14, m18, M25, M112, m139 and m142 peptides). Total numbers of IFN-γ^+^ TNF-α^+^ peptide specific CD4 T cells from B6 and *Il10*^-/-^ mice are shown. B) Splenocytes from infected mice were isolated at day 5.5 post infection. Total numbers of CD11c^+^MHCII^+^B220^-^ DCs (upper row) and CD11c^+^CD11b^+^MHCII^+^B220^-^ DCs (lower row) from infected B6 and *Il10*^-/-^ mice are shown (n=3). C) Expression levels of costimulatory molecules CD80, CD86 and CD40 on day 5.5 p.i. Plots are gated on CD11c^+^CD8α^+^MHCII^+^B220^-^ DCs (n=3, error bars indicate standard deviation). Representative FACS plots (left column) and summary of MFI data (right column) are shown. Statistical analysis was performed by 2-tailed unpaired student's t-test (* p<0.05, ** p<0.01, *** p<0.001).
