# Supplementary material for: IL-10 Suppression of NK/DC Crosstalk Leads to Poor Priming of MCMV-Specific CD4 T Cells and Prolonged MCMV Persistence
Source: PLoS Pathog. 2012 Aug 2;8(8):e1002846. doi: 10.1371/journal.ppat.1002846 (PMC3410900; doi:10.1371/journal.ppat.1002846)
Supplement: Figure S11 — IL-10 dampens DC/NK cross-talk during MCMV infection. (DOC) [file ppat.1002846.s011.doc]

**
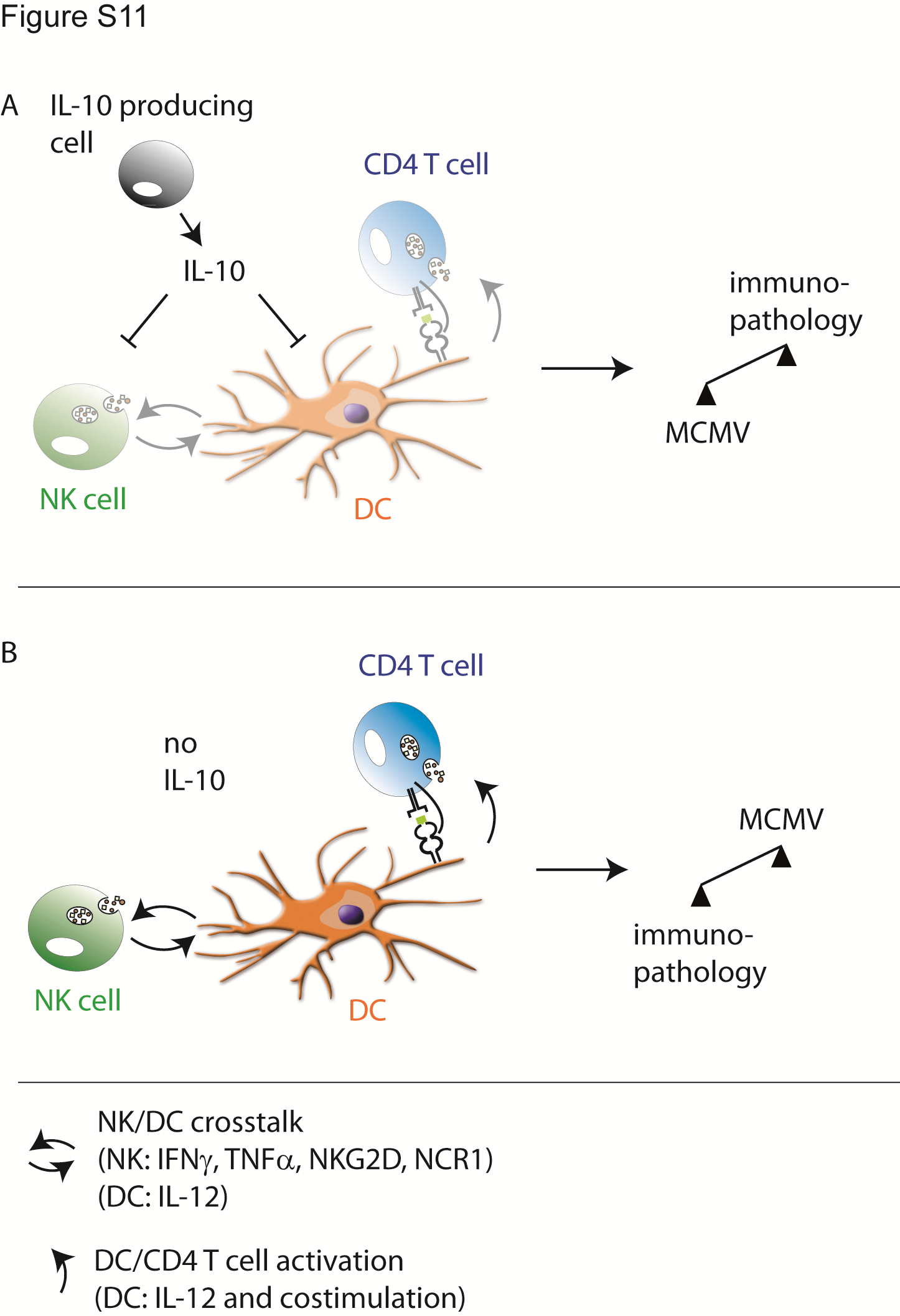
**

**Figure S11 IL-10 dampens DC/NK cross-talk during MCMV infection**

A) MCMV infection leads to early and robust IL-10 production during acute MCMV infection in B6 mice which impairs DC/NK cross talk, resulting in impaired priming of MCMV-specific CD4 T cells. This impaired DC/NK cross talk is restricted on the DC side to myeloid CD11b^+^ DCs (evidenced by reduced costimulatory molecule expression, reduced IL-12 production and impaired priming capacities for naive MCMV-specific CD4 T cells) and on the NK side is manifested by strongly impaired TNF-α and IFN-γ production. In consequence, MCMV-specific CD4 T cell responses are only poorly induced in B6 mice, leading to prolonged lytic viral replication but at the same time limited immunopathology.

B) In absence of IL-10, the full potential of NK/DC cross-talk is unleashed, with NK cells promoting DC activation via IFN-γ, TNF-α secretion and through NKG2D and NCR-1 engagement. Such fully activated DCs are superior in priming naive MCMV-specific CD4 T cell responses due to increased expression of costimulatory molecules and additional provision of the Th1 promoting cytokine IL-12. Consequently, MCMV-specific CD4 T cell responses are potently induced in *Il10*^-/-^ mice, mediating swift control of lytic viral replication, but at the expense of TNF-α-mediated immunopathology.
