# Supplementary material for: IL-10 Suppression of NK/DC Crosstalk Leads to Poor Priming of MCMV-Specific CD4 T Cells and Prolonged MCMV Persistence
Source: PLoS Pathog. 2012 Aug 2;8(8):e1002846. doi: 10.1371/journal.ppat.1002846 (PMC3410900; doi:10.1371/journal.ppat.1002846)
Supplement: Text S1 — Materials and methods for supplementary figures. (DOC) [file ppat.1002846.s012.doc]

**Text S1 Materials and Methods for supplementary figures**

**Antibodies**

The following fluorochrome-conjugated antibodies were obtained either from Biolegend (Lucerna Chem AG, Luzern, Switzerland) or from BD Biosciences (Allschwil, Switzerland): anti-CD4, anti-CD8, anti-CD3ε, anti TCRVα11.1, anti-TNF−α, anti-IFN-γ, anti-CD45.1.

**Generation and characterization of bone marrow chimeras**

*Il10rβ*^-/-^ mice were total body γ irradiated (950 rad) and reconstituted with a 1:1 mixture of bone marrow from *Il10rβ*^-/-^, C57BL/6 and *Cd4*^-/-^ mice. In other experiments *Il10*^-/-^ mice were total body γ irradiated (950 rad) and reconstituted with a 1:1 mixture of bone marrow from or CD11c-DTR/GFP, LysMCre/iDTR, C57BL/6 and *Cd4*^-/-^ mice. During the first 2 weeks of reconstitution, mice were treated with 1:250 dilution of 24% Borgal (Intervet, Boxmeer, Netherlands) in drinking water. Bone marrow chimeras were used for experiments 8-10 weeks after reconstitution. For CD11c^+^ cell and LysM^+^ cell depletions, test chimeras received i.p. injections of 1 μg diphtheria toxin (Sigma-Aldrich) every 2 days for a total period of 14 days.

**Generation of M25-II transgenic mouse line**

CD4 T cell hybridoma 10.18 B10 with specificity for the M25_aa410–425_ peptide of MCMV was previously described . RNA was isolated from 10.18 B10 hybridoma by RNeasy mini kit according to manufacturer’s instruction (Qiagen AG). Using primer sets specific for TCRα and TCRβ gene fragments ; cDNA was generated by SuperScript II reverse transcriptase according to the manufacturer’s instruction (Invitrogen, Basel, Switzerland). Obtained PCR products were sequenced by Microsynth (Zurich, Switzerland). The alignment of the obtained sequences to the mouse genome was done using Ensemble database (http:/www.ensembl.org/Mus_musculus). The identified TCR Vα11Jα30 and TCRVβ1Jβ2.5 gene fragments were amplified from genomic mouse DNA by PCR using the following primer sets: TCRVαfwd (5'-TTACCCGGGAGCGATTGGACAGGGGCCATG-3') and TCRVαrev (5'-ATATGCGGCCGCCTTCAGACCCACCTGGTTGCAC-3'); TCRVβfwd (5'-GCC CTC GAG AAT ACC CGT CTG GAG CCT GAT TCC ACC ATG-3') and TCRVβrev (5'-ATA CCG CGG AGA ACG CGC ACG TGG GGC CCC AGC TCA CCT A-3').

TCRVα11Jα30 and TCRVβ1Jβ2.5 gene fragments were digested with restriction endonucleases XmaI and NotI (for Vα11Jα30 gene fragment) respectively XhoI and SacII (for TCRVβ1Jβ2.5 gene fragments) and ligated into TCR expression cosmids . The obtained pTα Vα11Jα30 and pTβ Vβ1Jβ2.5 cosmids were digested with SalI and KpnI respectively in order to isolate the gene fragments from bacterial vector DNA. Linearized TCRVα11Jα30 and TCRVβ1Jβ2.5 gene fragments were then co-injected in equimolar ratios into fertilized C57BL/6N oocytes according to standard procedure . The obtained TCR transgenic mouse line were named according to the standardized genetic nomenclature for mice: C57BL/6N-*Tg(TcraM25,TcrbM25)424Biat* (M25-II) .

**CD4 T cell proliferation assay**

M25-II cells were isolated from splenocytes of naive M25-II transgenic mice by MACS positive selection with anti-CD4 microbeads (Miltenyi Biotech). Cells were labeled with 0.5μM CFSE (Invitrogen) for 8 min at 37ºC. Splenocytes were isolated from naive B6 mice. 1x10^5^ splenocytes were loaded with graded concentrations of M25 peptide and co-cultured with 6x10^4^ CFSE labeled M25-II cells. After 3 days of incubation, cells were stained with anti-CD4 and anti-CD45.1 antibodies and CFSE dilution was measured by flow cytometry.

**References**

1. Walton SM, Wyrsch P, Munks MW, Zimmermann A, Hengel H, et al. (2008) The dynamics of mouse cytomegalovirus-specific CD4 T cell responses during acute and latent infection. J Immunol 181: 1128-1134.

2. Yoshida R, Yoshioka T, Yamane S, Matsutani T, Toyosaki-Maeda T, et al. (2000) A new method for quantitative analysis of the mouse T-cell receptor V region repertoires: comparison of repertoires among strains. Immunogenetics 52: 35-45.

3. Pannetier C, Cochet M, Darche S, Casrouge A, Zoller M, et al. (1993) The sizes of the CDR3 hypervariable regions of the murine T-cell receptor beta chains vary as a function of the recombined germ-line segments. Proc Natl Acad Sci U S A 90: 4319-4323.

4. Kouskoff V, Signorelli K, Benoist C, Mathis D (1995) Cassette vectors directing expression of T cell receptor genes in transgenic mice. J Immunol Methods 180: 273-280.

5. Rulicke T (2004) Pronuclear microinjection of mouse zygotes. Methods Mol Biol 254: 165-194.

6. Blake JA, Bult CJ, Kadin JA, Richardson JE, Eppig JT (2011) The Mouse Genome Database (MGD): premier model organism resource for mammalian genomics and genetics. Nucleic Acids Res 39: D842-848.
